# Supplementary material for: A re-appraisal of the ENSO response to volcanism with paleoclimate data assimilation
Source: Nat Commun. 2022 Feb 8;13:747. doi: 10.1038/s41467-022-28210-1 (PMC8826427; doi:10.1038/s41467-022-28210-1)
Supplement: Supplementary file 1 — Supplementary Information [file 41467_2022_28210_MOESM1_ESM.pdf]

Supplementary Information for “A re-appraisal of the ENSO response to volcanism with paleoclimate data assimilation”

Feng Zhu[1,2], Julien Emile-Geay[2,*], Kevin J. Anchukaitis[3,4,5], Gregory J. Hakim[6], Andrew T. Wittenberg[7], Mariano S. Morales[8,9], Matthew Toohey[10], and Jonathan King[4,5]

[1]School of Atmospheric Sciences, Nanjing University of Information Science and Technology, Nanjing, China
[2]Department of Earth Sciences, University of Southern California, Los Angeles, CA USA
[3]Laboratory of Tree-Ring Research, University of Arizona, Tucson AZ USA
[4]School of Geography, Development, and Environment, University of Arizona, Tucson AZ USA
[5]Department of Geosciences, University of Arizona, Tucson AZ USA
[6]Department of Atmospheric Sciences, University of Washington, Seattle, WA USA
[7]NOAA Geophysical Fluid Dynamics Laboratory, Princeton, NJ, USA
[8]Instituto Argentino de Nivología, Glaciologa y Cs. Ambientales, Consejo Nacional de Investigaciones Cientficas y Técnicas (CONICET), 5500 Mendoza, Argentina
[9]Laboratorio de Dendrocronología, Universidad Continental, 12003 Huancayo, Perú
[10]Institute of Space and Atmospheric Studies, University of Saskatchewan, Saskatoon, Canada
[*]Corresponding author: julieneg@usc.edu

Inventory

- Supplementary Text 1-7
- Supplementary Tables 1-3
- Supplementary Figures 1-25

Contents

Supplementary Text 1: Cross-validation of the Last Millennium Reanalysis reconstructions 1
Supplementary Text 2: False positives in superposed epoch analysis 7
Supplementary Text 3: The ENSO response to volcanism in PMIP3 and CESM-LME simulations 8
Supplementary Text 4: Sensitivity to reconstruction season in the Last Millennium Reanalysis 12
Supplementary Text 5: A comparison of historical SST analyses 18
Supplementary Text 6: Investigating nonstationarity 20
Supplementary Text 7: Exploring discrepancies 24
References 27

Supplementary Text 1: Cross-validation of the Last Millennium Reanalysis reconstructions

The Last Millennium Reanalysis (LMR)[1,2] reconstructions in this study show high skill compared to many other existing reconstructions (Supplementary Fig. 1), yet they are performed with a calibration period over 1850-2000 CE, which overlaps with the validation period (1881-2000 CE) and leads to concerns of potential overfitting.

To evaluate whether overfitting actually occurs, we repeat the LMR reconstructions described in the main text, LMR (Corals), LMR (Li13b6), and LMR (Corals+Li13b6), with calibration period over 1881-1940 CE and 1941-2000 CE. For comparison, we repeat the validation in Fig. 1 (main text) over 1881-1940 CE (Supplementary Fig. 2) and 1941-2000 CE (Supplementary Fig. 3) as baselines (tagged as Test I and II, respectively). The reconstruction with calibration period over 1941-2000 CE is verified over validation period 1881-1940 CE (tagged as Test III) (Supplementary Fig. 4), and the

reconstruction with calibration period over 1981-1940 CE is verified over validation period 1941-2000 CE (tagged as Test IV) (Supplementary Fig. 5), so that the calibration and validation periods are disjoint.

The validation skill of the reconstructed Niño 3.4 is summarized in Supplementary Table 1. Comparing Test III with Test I, the coefficient of determination (R^2) drops by 0.01, 0.08, and 0.05 for LMR (Corals), LMR (Li13b6), and LMR (Corals+Li13b6), respectively, while the coefficient of efficiency (CE)[3] drops by -0.08, 0.10, and 0.05, respectively. Similarly, comparing Test IV with Test II, R^2 drops by 0.01, 0.08, and 0.03 for LMR (Corals), LMR (Li13b6), and LMR (Corals+Li13b6), respectively, while CE drops by 0.09, 0.11, and 0.04, respectively. Overall, the skill degradation is at an acceptable level, and we conclude that overfitting is not a major issue with the original reconstructions in the main text. It is also worth noting that LMR (Corals+Li13b6) performs the best in all of the tests compared to LMR (Corals) and LMR (Li13b6), indicating that the strategy of combining both tree-ring and coral archives is working as expected.

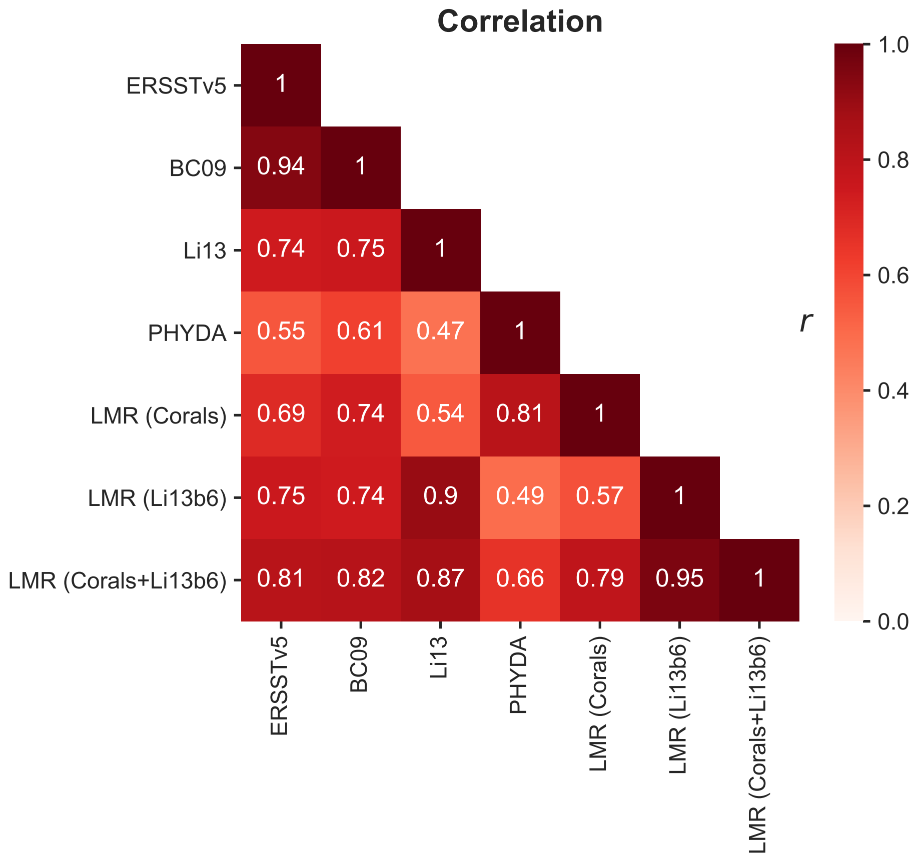

Supplementary Fig. 1. Correlation coefficient between instrumental observations and reconstructions of December-February (DJF) Niño 3.4 over timespan 1881-2000 CE. Data sources include: Extended Reconstructed Sea Surface Temperature, Version 5 (ERSSTv5)[4], Bunge and Clarke[5] (denoted as BC09), Li et al.[6] (denoted as Li13), the Paleo Hydrodynamics Data Assimilation product (PHYDA)[7], and the Last Millennium Reanalysis (LMR)[1,2] reconstructions of this study: LMR (Corals), LMR (Li13b6), and LMR (Corals+Li13b6). Note that Li13 is a November-Januray (NDJ) reconstruction, and its correlation to NDJ ERSSTv5 and NDJ BC09 is 0.76 and 0.75, respectively.

Supplementary Table 1. Validation skill of the Last Millennium Reanalysis (LMR)[1,2] Niño 3.4 reconstructions.

Test	Calibration period	Validation period	LMR (Corals)	LMR (Li13b6)	LMR (Corals+Li13b6)
I	1850-2000	1881-1940	$R^2 = 0.43, CE = 0.35$	$R^2 = 0.62, CE = 0.62$	$R^2 = 0.66, CE = 0.65$
II	1850-2000	1941-2000	$R^2 = 0.53, CE = 0.52$	$R^2 = 0.52, CE = 0.51$	$R^2 = 0.65, CE = 0.64$
III	1941-2000	1881-1940	$R^2 = 0.42, CE = 0.40$	$R^2 = 0.54, CE = 0.52$	$R^2 = 0.61, CE = 0.60$
IV	1881-1940	1941-2000	$R^2 = 0.52, CE = 0.43$	$R^2 = 0.44, CE = 0.40$	$R^2 = 0.62, CE = 0.60$

R^2=coefficient of determination, CE=coefficient of efficiency[3].

Test I

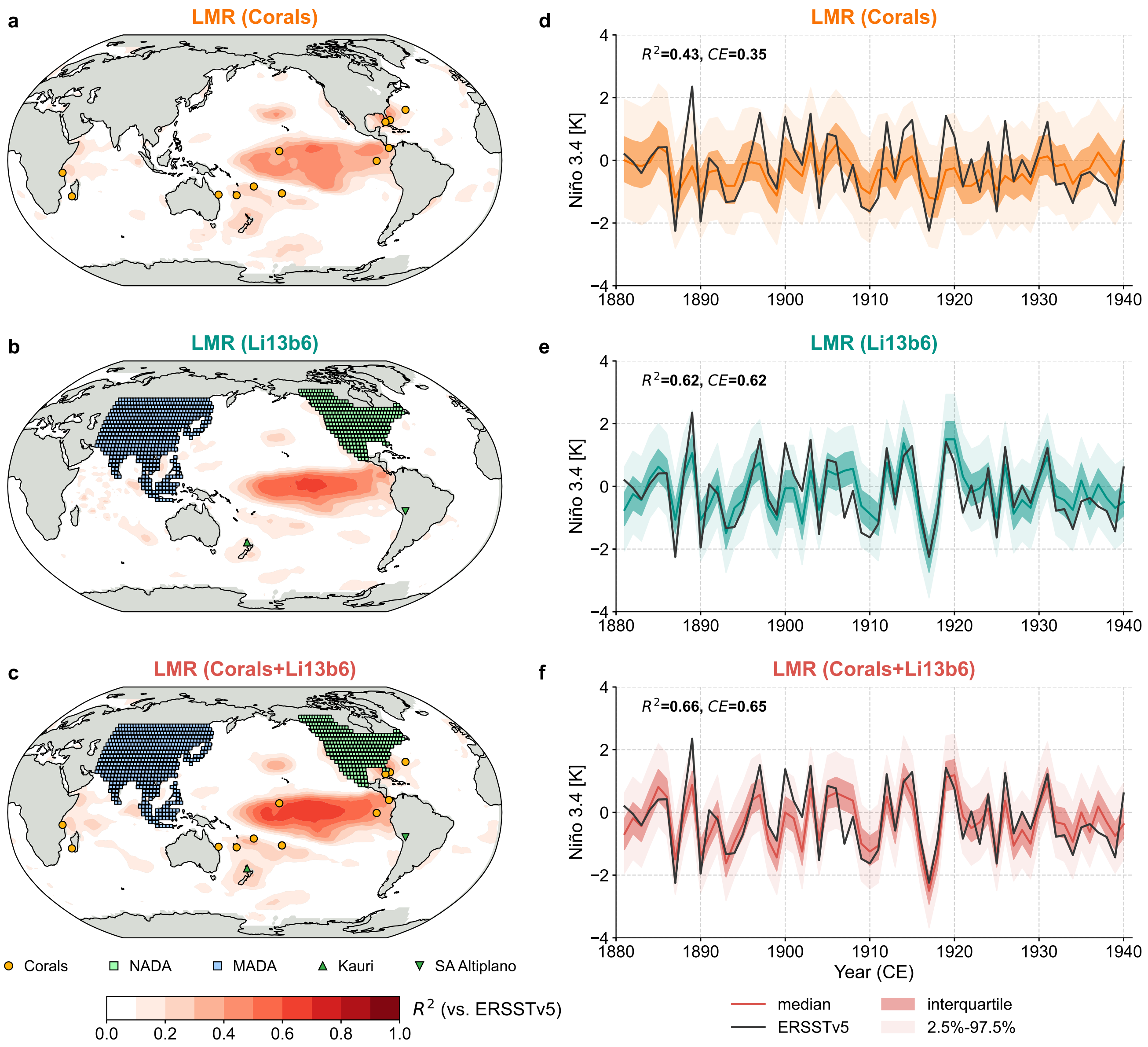

Supplementary Fig. 2. Validation of the Last Millennium Reanalysis (LMR)[1,2] sea surface temperature reconstructions over 1881-1940 CE, with calibration period 1850-2000 CE. The labels LMR (Corals), LMR (Li13b6), and LMR (Corals+Li13b6) denote the reconstructions assimilating corals reaching back before 1750 CE from the Ocean2k compilation[8] updated with the latest Palmyra data[9], the six best predictors from Li et al.[6] (denoted as Li13b6), and both data sources, respectively. **(a-c)** Spatial verification of the median field of the reconstructed boreal winter (December-February, DJF) surface temperature. Validation is performed against the Extended Reconstructed Sea Surface Temperature, Version 5 (ERSSTv5)[4]. The orange dots denote the location of the corals, the mint and blue squares denote the location of the North American Drought Atlas (NADA) (Version 2a)[10] and Monsoon Asia Drought Atlas (MADA)[11] sites, the green upward triangle denotes the location of the Kauri tree-ring composite[12], and the green downward triangle denotes the location of the South America Altiplano (SA Altiplano) tree-ring composite[13]. **(d-f)** Temporal verification of the median of the LMR reconstructed DJF Niño 3.4 series (colored curves) against the ERSSTv5 derived Niño 3.4 (black solid curve). For each reconstruction, dark shading denotes the interquartile range, and light shading denotes the central 95% region, from 2.5% to 97.5%. R^2=coefficient of determination, CE=coefficient of efficiency[3].

Test II

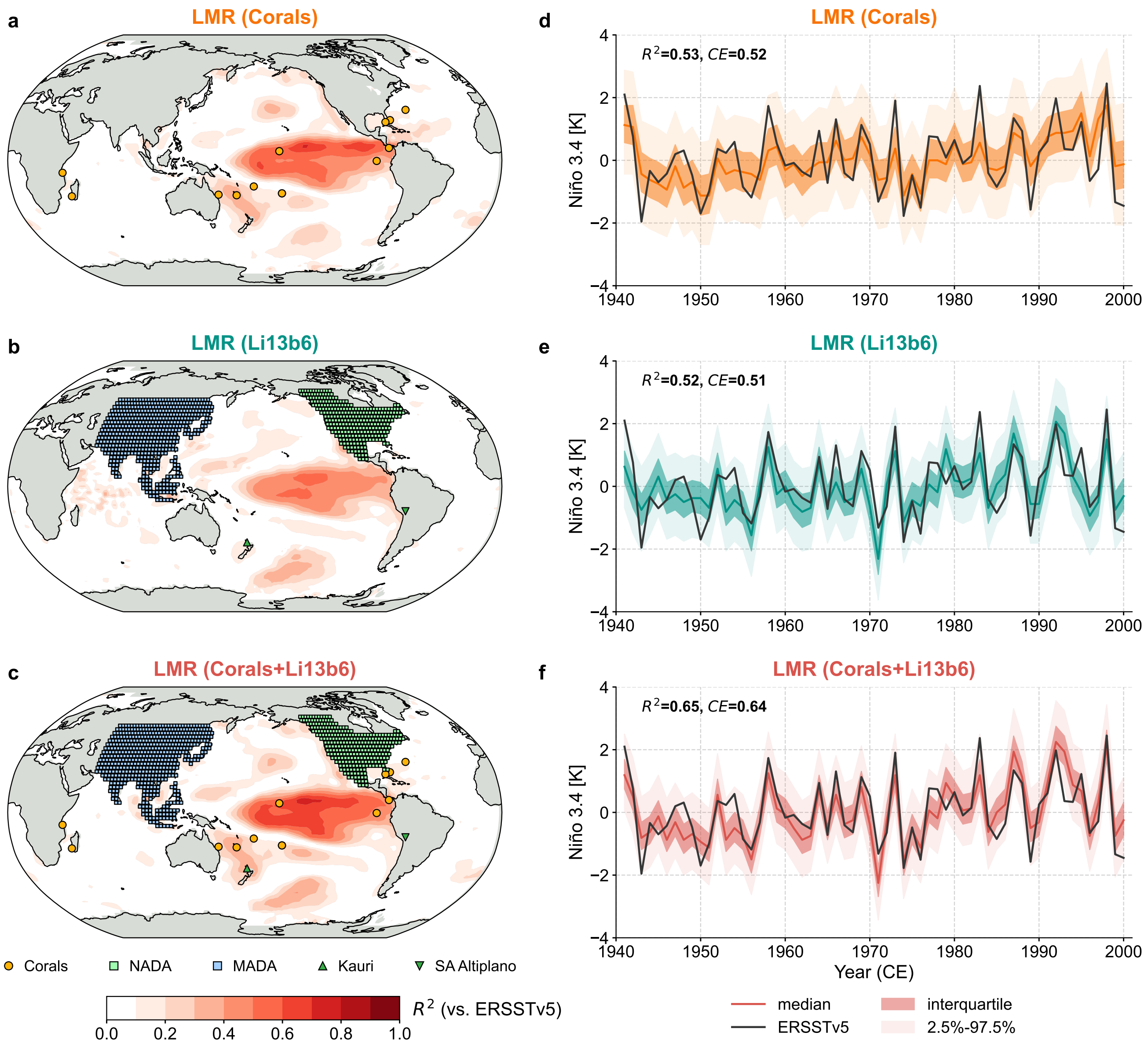

Supplementary Fig. 3. Validation of the Last Millennium Reanalysis (LMR)[1,2] sea surface temperature reconstructions over 1941-2000 CE, with calibration period 1850-2000 CE. The labels LMR (Corals), LMR (Li13b6), and LMR (Corals+Li13b6) denote the reconstructions assimilating corals reaching back before 1750 CE from the Ocean2k compilation[8] updated with the latest Palmyra data[9], the six best predictors from Li et al.[6] (denoted as Li13b6), and both data sources, respectively. **(a-c)** Spatial verification of the median field of the reconstructed boreal winter (December-February, DJF) surface temperature. Validation is performed against the Extended Reconstructed Sea Surface Temperature, Version 5 (ERSSTv5)[4]. The orange dots denote the location of the corals, the mint and blue squares denote the location of the North American Drought Atlas (NADA) (Version 2a)[10] and Monsoon Asia Drought Atlas (MADA)[11] sites, the green upward triangle denotes the location of the Kauri tree-ring composite[12], and the green downward triangle denotes the location of the South America Altiplano (SA Altiplano) tree-ring composite[13]. **(d-f)** Temporal verification of the median of the LMR reconstructed DJF Niño 3.4 series (colored curves) against the ERSSTv5 derived Niño 3.4 (black solid curve). For each reconstruction, dark shading denotes the interquartile range, and light shading denotes the central 95% region, from 2.5% to 97.5%. R^2=coefficient of determination, CE=coefficient of efficiency[3].

Test III

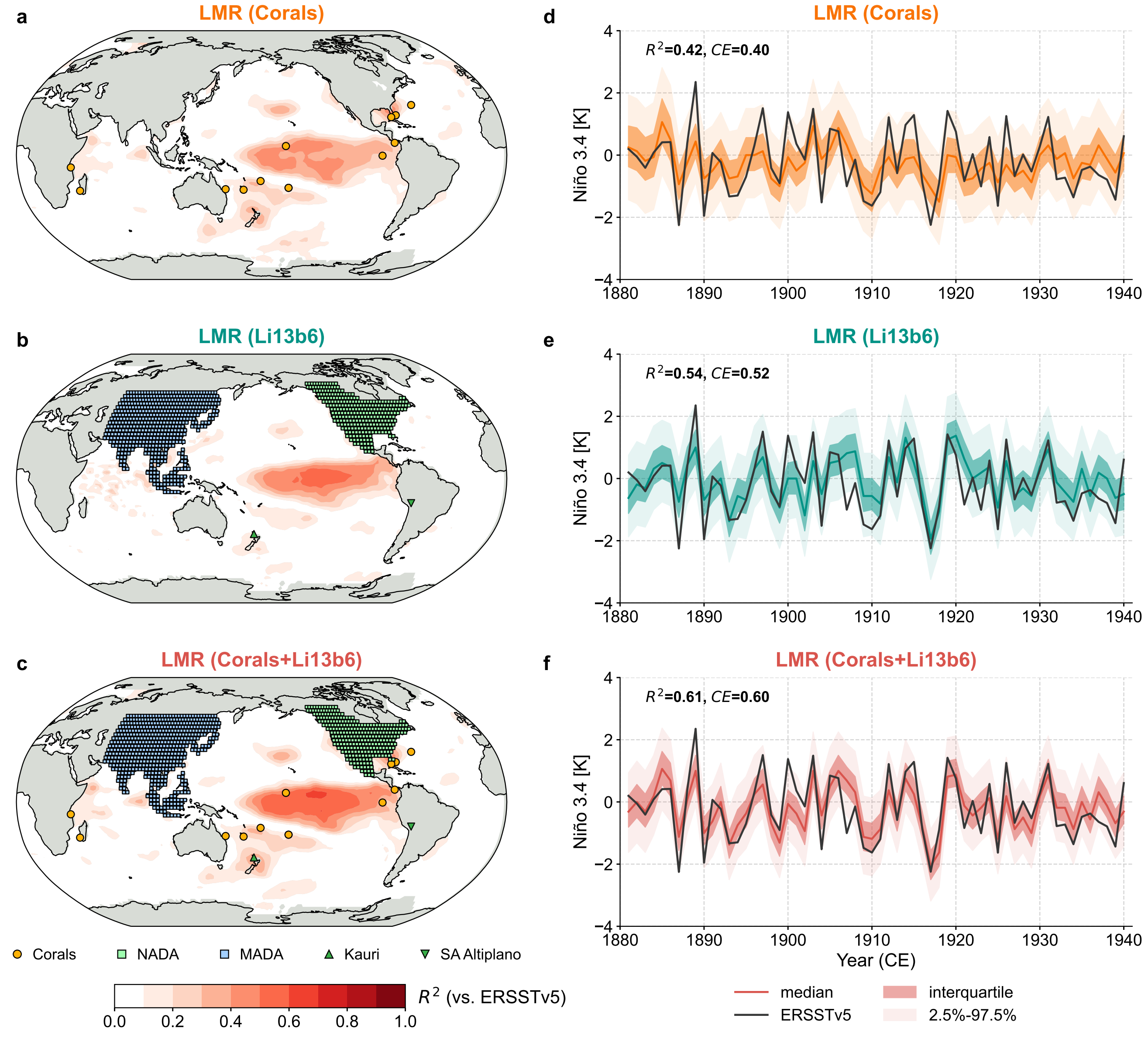

Supplementary Fig. 4. Validation of the Last Millennium Reanalysis (LMR)[1,2] sea surface temperature reconstructions over 1881-1940 CE, with calibration period 1941-2000 CE. The labels LMR (Corals), LMR (Li13b6), and LMR (Corals+Li13b6) denote the reconstructions assimilating corals reaching back before 1750 CE from the Ocean2k compilation[8] updated with the latest Palmyra data[9], the six best predictors from Li et al.[6] (denoted as Li13b6), and both data sources, respectively. **(a-c)** Spatial verification of the median field of the reconstructed boreal winter (December-February, DJF) surface temperature. Validation is performed against the Extended Reconstructed Sea Surface Temperature, Version 5 (ERSSTv5)[4]. The orange dots denote the location of the corals, the mint and blue squares denote the location of the North American Drought Atlas (NADA) (Version 2a)[10] and Monsoon Asia Drought Atlas (MADA)[11] sites, the green upward triangle denotes the location of the Kauri tree-ring composite[12], and the green downward triangle denotes the location of the South America Altiplano (SA Altiplano) tree-ring composite[13]. **(d-f)** Temporal verification of the median of the LMR reconstructed DJF Niño 3.4 series (colored curves) against the ERSSTv5 derived Niño 3.4 (black solid curve). For each reconstruction, dark shading denotes the interquartile range, and light shading denotes the central 95% region, from 2.5% to 97.5%. R^2=coefficient of determination, CE=coefficient of efficiency[3].

Test IV

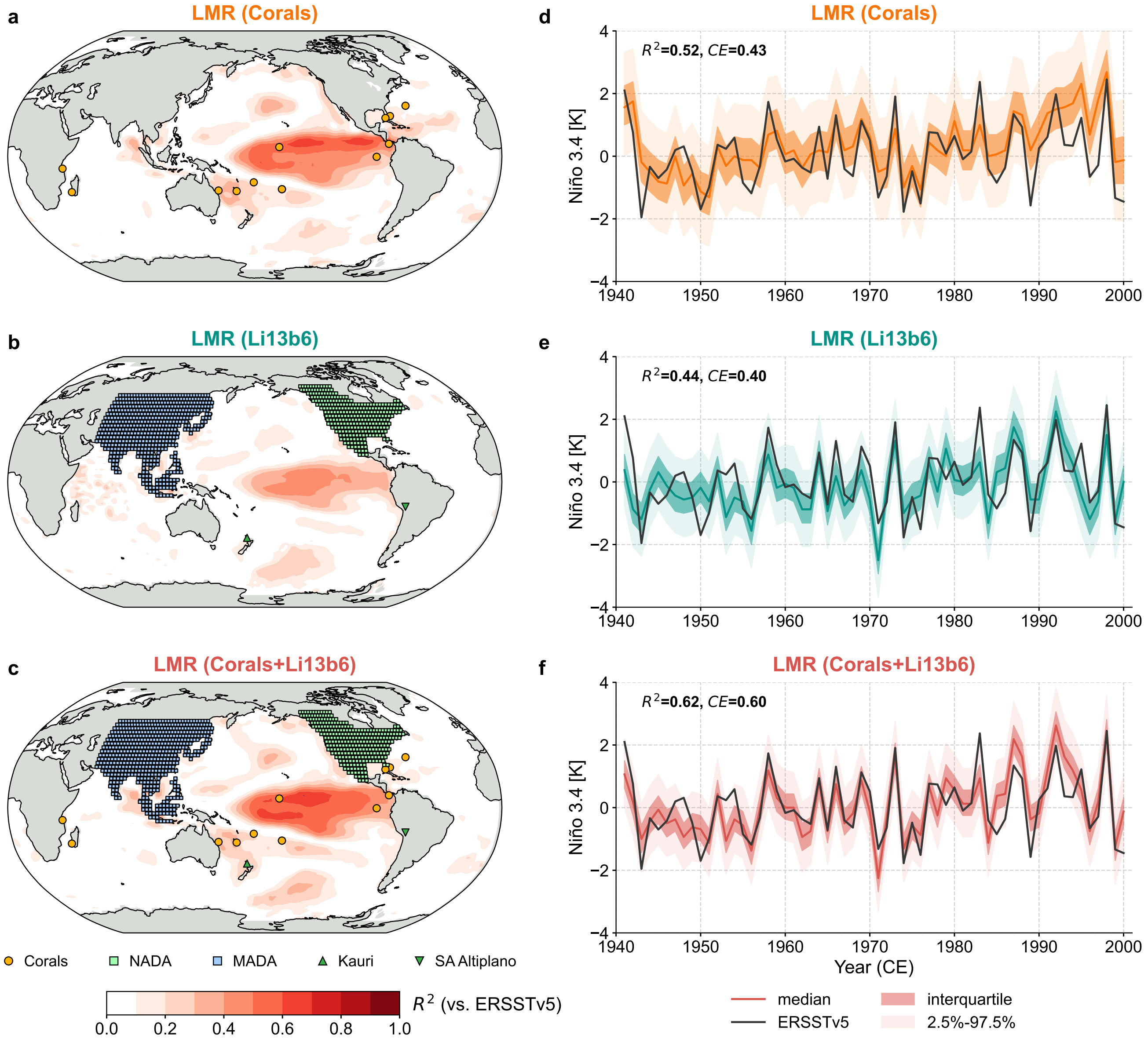

Supplementary Fig. 5. Validation of the Last Millennium Reanalysis (LMR)[1,2] sea surface temperature reconstructions over 1941-2000 CE, with calibration period 1881-1940 CE. The labels LMR (Corals), LMR (Li13b6), and LMR (Corals+Li13b6) denote the reconstructions assimilating corals reaching back before 1750 CE from the Ocean2k compilation[8] updated with the latest Palmyra data[9], the six best predictors from Li et al.[6] (denoted as Li13b6), and both data sources, respectively. **(a-c)** Spatial verification of the median field of the reconstructed boreal winter (December-February, DJF) surface temperature. Validation is performed against the Extended Reconstructed Sea Surface Temperature, Version 5 (ERSSTv5)[4]. The orange dots denote the location of the corals, the mint and blue squares denote the location of the North American Drought Atlas (NADA) (Version 2a)[10] and Monsoon Asia Drought Atlas (MADA)[11] sites, the green upward triangle denotes the location of the Kauri tree-ring composite[12], and the green downward triangle denotes the location of the South America Altiplano (SA Altiplano) tree-ring composite[13]. **(d-f)** Temporal verification of the median of the LMR reconstructed DJF Niño 3.4 series (colored curves) against the ERSSTv5 derived Niño 3.4 (black solid curve). For each reconstruction, dark shading denotes the interquartile range, and light shading denotes the central 95% region, from 2.5% to 97.5%. R^2=coefficient of determination, CE=coefficient of efficiency[3].

Supplementary Text 2: False positives in superposed epoch analysis

Superposed epoch analysis (SEA) is widely used for the analysis of ENSO response to volcanism[6,9,14], yet the sample size is usually small due to the infrequent nature of explosive eruptions. Therefore type I error (the probability of false positives) is potentially a risk of the analysis, and translates to accidentally identifying significant ENSO responses when they are in fact due to other factors (e.g. internal variability).

We assess this risk utilizing a 4000 year long simulation of the GFDL CM2.1 model[15,16]. The run employs stationary boundary conditions, and is therefore free of volcanic influences. Following the procedure of SEA described in the Methods section of the main text, we calculate the boreal winter (DJF) Niño 3.4 timeseries from the simulation, and convert it to year 1 Niño 3.4 anomaly, which is defined as the original value at each year 1 with the mean of three years prior to year 0 removed.

We define a positive event as the year when the composite of pre-defined volcanic years is larger than a certain quantile (80%, 90%, or 95%) of the composites of randomly drawn non-volcanic years, which is approximately the same quantile of all years, as we test only with a small number of "volcanic" years compared to the whole sample (4000 years). Since the simulation is free of volcanic forcing, there could be no causal relationship between volcanism and ENSO events, and any positive event is hence a misattribution.

In practice, we test the cases with $n = 3, 5, 10, 15, 20$ "event" years. For instance, when $n = 20$, we randomly draw 20 years as "event" years 10,000 times (with replacement). Each time, we calculate the composite of year 1 Niño 3.4 anomaly over volcanic years, compare it to the (80%, 90%, or 95%) quantiles of the composites of all years, and flag the event as positive when the composite value of volcanic years is larger than the (80%, 90%, or 95%) percentiles of all years. Then we compute the false positive rate (FPR) as the number of positive events divided by 10,000.

Supplementary Table 2. Probability of accidentally identifying significant ENSO response.

Sample size	FPR (80%)	FPR (90%)	FPR (95%)
$n = 3$	10.55%	1.6%	4×10^{-4}
$n = 5$	5.49%	0.33%	$< 1 \times 10^{-4}$
$n = 10$	1.21%	2×10^{-4}	$< 1 \times 10^{-4}$
$n = 15$	0.25%	$< 1 \times 10^{-4}$	$< 1 \times 10^{-4}$
$n = 20$	7×10^{-4}	$< 1 \times 10^{-4}$	$< 1 \times 10^{-4}$

FPR=false positive rate

Supplementary Table 2 shows the probability of accidentally identifying significant ENSO response in such a SEA setting. It can be seen that for the 95% level, the FPR is close to 0 even for a tiny sample size $n = 3$. For the permissive 80% level, the FPR is non-ignorable when $n \leq 5$, and but becomes acceptably small (close to 1%) when $n \geq 10$. Based on the results above, we conclude that SEA is overall robust to type I error; that is, the small sample size is not likely to cause a false detection of significant event in the composite. It is worth noting, however, that this does not imply that SEA is robust regarding other uncertainties, as discussed in the main text.

Supplementary Text 3: The ENSO response to volcanism in PMIP3 and CESM-LME simulations

Previous modeling studies have suggested overall significant ENSO response to volcanism[17,18]. Here we investigate the ENSO response to volcanism in Paleoclimate Modelling Intercomparison Project Phase III (PMIP3)[19] and Community Earth System Model Last Millennium Ensemble (CESM-LME) simulations[20] with SEA. Since two different volcanic forcing sources[21,22] are applied in these simulations (Supplementary Table 3), we compare these forcing sources with eVolv2k v3[23] that we used for the analysis for reconstructions, and select eruption events that are in agreement but only with small temporal offsets for our analysis (Supplementary Fig. 6). The results of SEA applied on SST based Niño 3.4 anomaly values are shown in Supplementary Fig. 7, 8, and the same analyses applied on relative sea surface temperature (RSST)[24] based Niño 3.4 anomaly values are shown in Supplementary Fig. 9, 10. The difference between SST and RSST in individual simulations are shown in Supplementary Fig. 11.

Comparing the SST based and RSST based results, we confirm the impact of the RSST conversion on the analyses as suggested in previous studies[24,25], which overall boosts the significance of ENSO response to volcanism. This impact is not obvious in our reconstruction (Supplementary Fig. 12) due to the sparse geographical coverage of the proxies, as discussed in the main text. However, we note that, even with the RSST conversion, insignificant year 1 responses are still observed in simulations such as BCC, FGOALS, IPSL, and MIROC in SEA.

Supplementary Table 3. Volcanic forcing applied in Paleoclimate Modelling Intercomparison Project Phase III (PMIP3)[19] and Community Earth System Model Last Millennium Ensemble (CESM-LME) simulations[20].

Model	BCC	CCSM4	MRI	CESM	FGOALS	IPSL	GISS	CISRO	HadCM3	MIROC	MPI
Forcing	GRA	GRA	GRA	GRA	GRA	GRA	GRA	CEA	CEA	CEA	CEA

GRA = Gao et al. (2008)[21]; CEA = Crowley et al. (2008)[22].

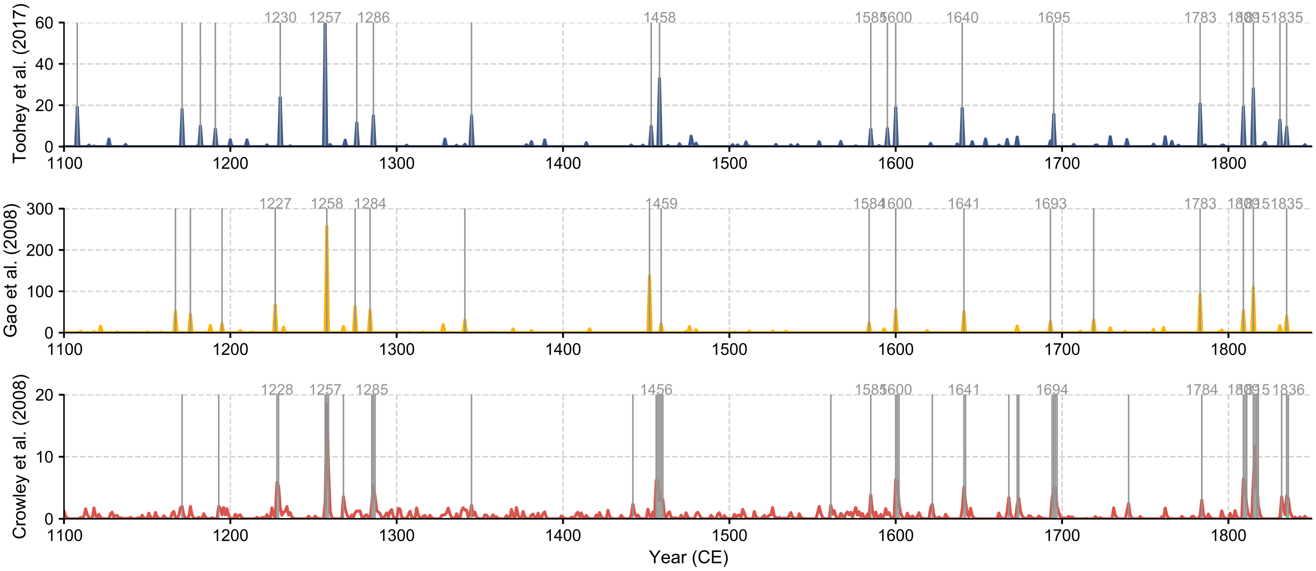

Supplementary Fig. 6. The three volcanic forcing sources in timeseries: Toohey et al. (2017)[23], Gao et al. (2008)[21], and Crowley et al. (2008)[22]. Vertical grey curves denote the years with relative large forcing values (> 6, > 20, > 2, respectively), among which the consistent events (with small temporal offsets) detected in all of the three sources are labeled with the year. The quantities of the three sources are global volcanic stratospheric sulfur injection of eruption, global total stratospheric sulfate aerosol injection, and averaged extratropical aerosol optical depth, respectively.

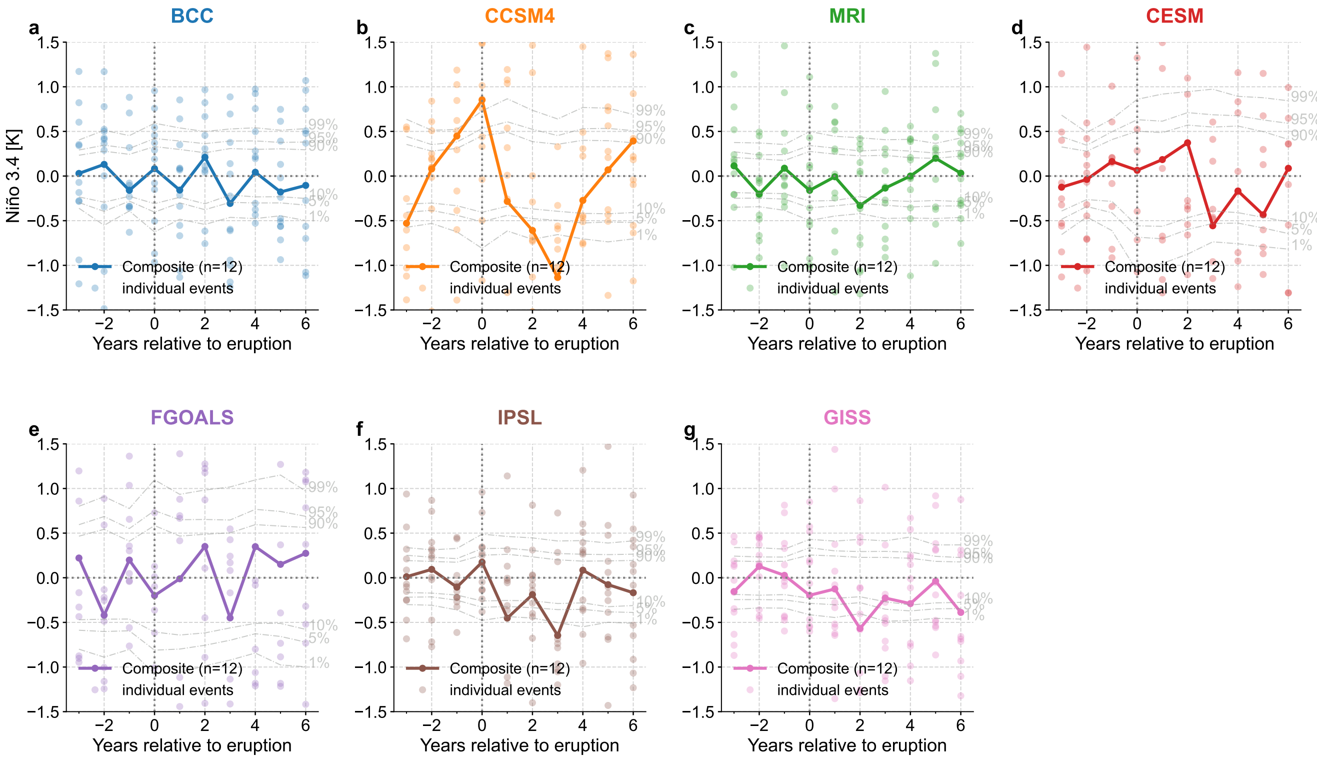

Supplementary Fig. 7. Superposed epoch analysis (SEA) of the sea surface temperature (SST) based Niño 3.4 model simulations with Gao et al. (2008)[21] volcanic forcing applied. Solid curves with dark dots denote the composite mean, and the light dots denote the Niño 3.4 anomaly at each year for each individual event. The light grey dashed curves denote the 1%, 5%, 10%, 90%, 95%, and 99% quantiles of the composite means from 1000 bootstrap draws from non-volcanic years (see Methods for the details).

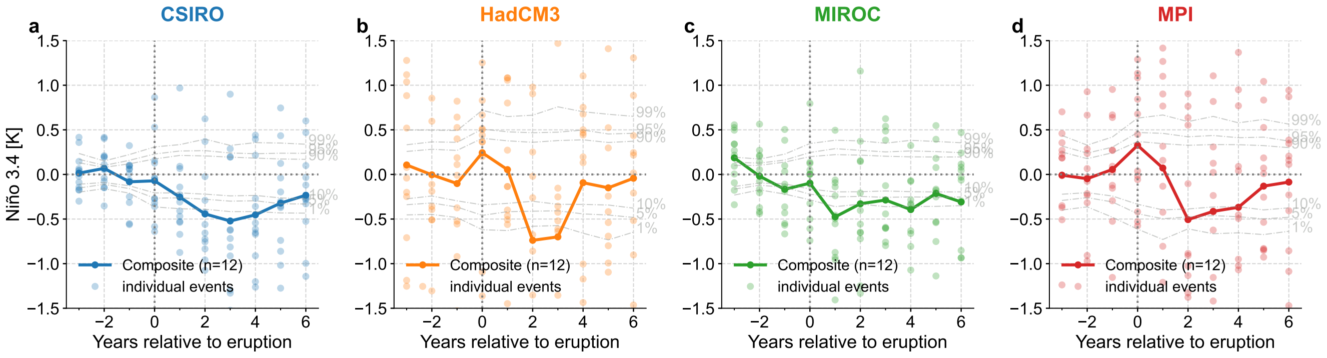

Supplementary Fig. 8. Superposed epoch analysis (SEA) of the sea surface temperature (SST) based Niño 3.4 model simulations with Crowley et al. (2008)[22] volcanic forcing applied. Solid curves with dark dots denote the composite mean, and the light dots denote the Niño 3.4 anomaly at each year for each individual event. The light grey dashed curves denote the 1%, 5%, 10%, 90%, 95%, and 99% quantiles of the composite means from 1000 bootstrap draws from non-volcanic years (see Methods for the details).

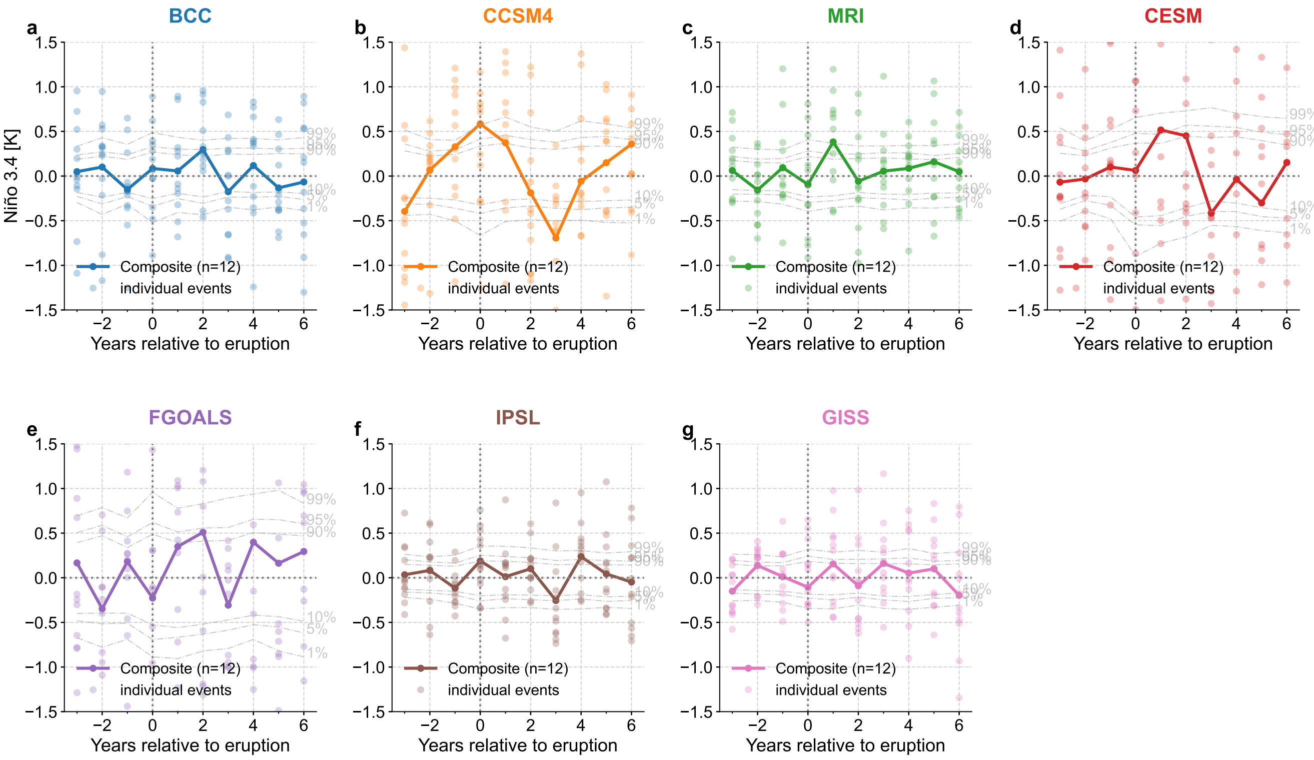

Supplementary Fig. 9. Superposed epoch analysis (SEA) of the relative sea surface temperature (RSST) based Niño 3.4 model simulations with Gao et al. (2008)[21] volcanic forcing applied. Solid curves with dark dots denote the composite mean, and the light dots denote the Niño 3.4 anomaly at each year for each individual event. The light grey dashed curves denote the 1%, 5%, 10%, 90%, 95%, and 99% quantiles of the composite means from 1000 bootstrap draws from non-volcanic years (see Methods for the details).

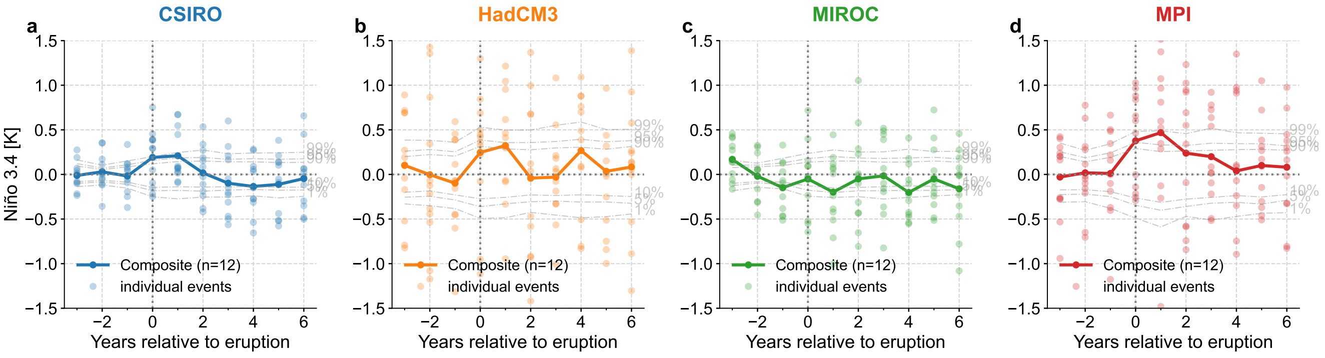

Supplementary Fig. 10. Superposed epoch analysis (SEA) of the relative sea surface temperature (RSST) based Niño 3.4 model simulations with Crowley et al. (2008)[22] volcanic forcing applied. Solid curves with dark dots denote the composite mean, and the light dots denote the Niño 3.4 anomaly at each year for each individual event. The light grey dashed curves denote the 1%, 5%, 10%, 90%, 95%, and 99% quantiles of the composite means from 1000 bootstrap draws from non-volcanic years (see Methods for the details).

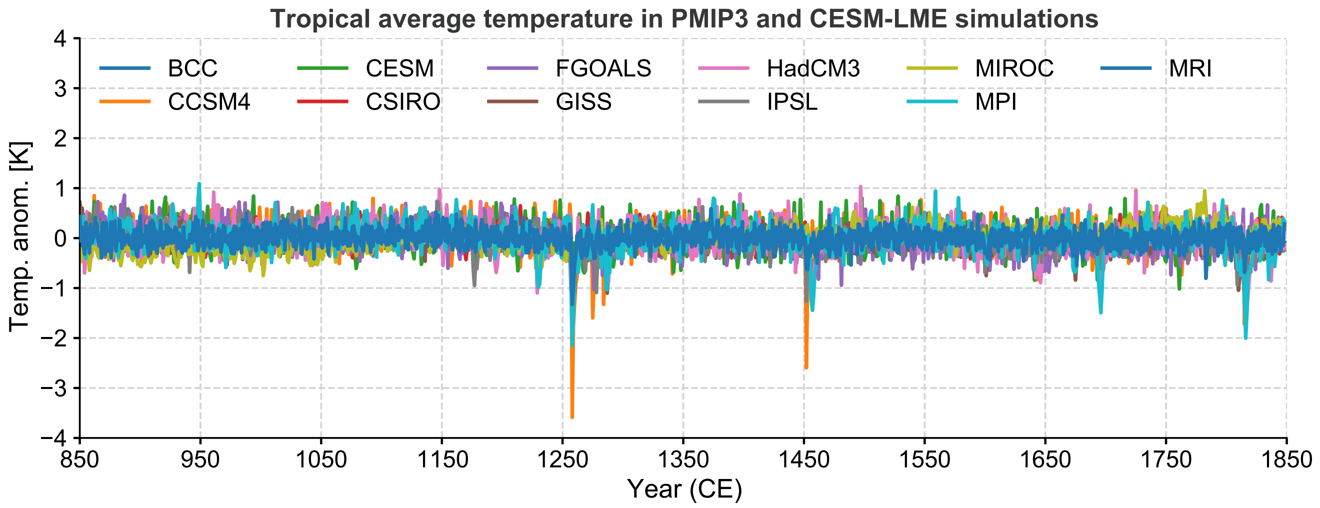

Supplementary Fig. 11. Tropical average temperature [the difference between sea surface temperature (SST) and relative sea surface temperature (RSST)] in PMIP3 and CESM-LME simulations.

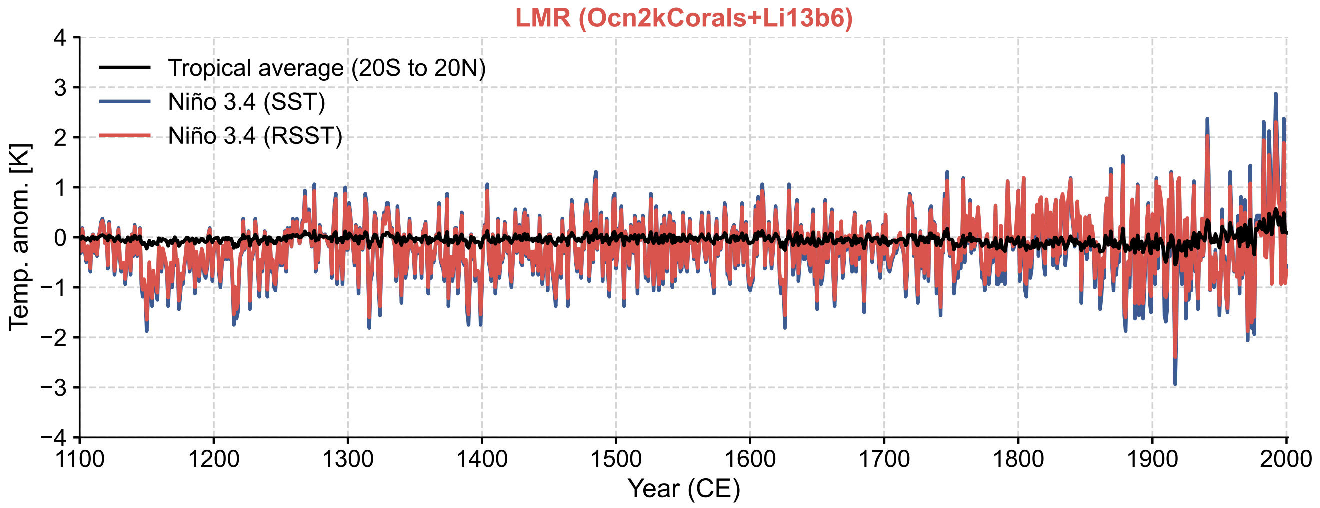

Supplementary Fig. 12. The timeseries of tropical average (20S-20N) temperature anomaly, sea surface temperature (SST) based Niño 3.4, and relative sea surface temperature (RSST)[24] based Niño 3.4 in the Last Millennium Reanalysis (LMR) reconstruction. LMR (Ocn2kCorals+Li13b6) denotes the reconstruction assimilating the whole coral collection in Ocean2k[8, 26] and the six best ENSO predictors from Li et al.[6]. We assimilate the whole coral collection here to better reconstruct the tropical Pacific region so that the tropical average temperature anomaly can be better estimated, at least over the instrumental era.

Supplementary Text 4: Sensitivity to reconstruction season in the Last Millennium Reanalysis

In the main text, the reconstruction season is December-February (DJF), when the ENSO signal is most strongly expressed[27]. Yet, a recent modeling study[28] has suggested that the ENSO response to volcanism is rather weak during DJF, but could be strong during January-September (JAS) and/or October-December (OND). Therefore, we repeat our reconstruction experiments for these two seasons along with the SEA to test the sensitivity of our conclusion to the choice of the reconstruction season. First, we evaluate the correlation between the best six predictors in Li et al.[6] (Li13b6) and the Niño 3.4 signal in the Extended Reconstructed Sea Surface Temperature v5 (ERSSTv5)[4]. Supplementary Fig. 13 presents a benchmark against the DJF Niño 3.4 signal, while Supplementary Fig. 14 and Supplementary Fig. 15 show evaluation against the JAS and OND Niño 3.4 signals, respectively. It can be seen that OND is overall a better target season than JAS, although the correlation skill with the JAS target is still acceptable. Therefore, we may treat Li13b6 as reliable predictors for both the JAS and OND ENSO signal. In addition, since most coral records used for the reconstruction are monthly resolved, they can be processed to reconstruct the JAS and OND seasons without seasonal bias.

Supplementary Fig. 16 and Supplementary Fig. 17 indicate that the reconstruction skill for the JAS and OND seasons is comparable to that with a DJF reconstruction season. Specifically, the skill for the JAS reconstruction is slightly weaker due to the worse correlation between Li13b6 and the JAS ENSO signal (Supplementary Fig. 14), while the skill for the OND reconstruction is slightly better than that with a DJF target due to the slightly better correlation between Li13b6 and the OND ENSO signal (Supplementary Fig. 15). Therefore, the reconstructions for those three seasons are comparably successful.

With the SEA on LMR (Corals+Li13b6) using the JAS and OND target seasons (Supplementary Fig. 18), we see an overall similar pattern, but weaker post-eruption responses compared to the DJF reconstruction, with a backward 1-year shift, leaving the year 1 response peaking. This is because the labeling of the time axis of the reconstructed Niño 3.4 series follows the year of January for the DJF reconstruction, while for the JAS/OND reconstructions, the labeling follows the year of July/October. This result indicates two points:

- Our conclusion is insensitive to the choice of the reconstruction season
- As discussed in the main text, the SEA method is sensitive to the dating uncertainty in either the reconstruction or the volcanic forcing, calling for caution on such analysis.

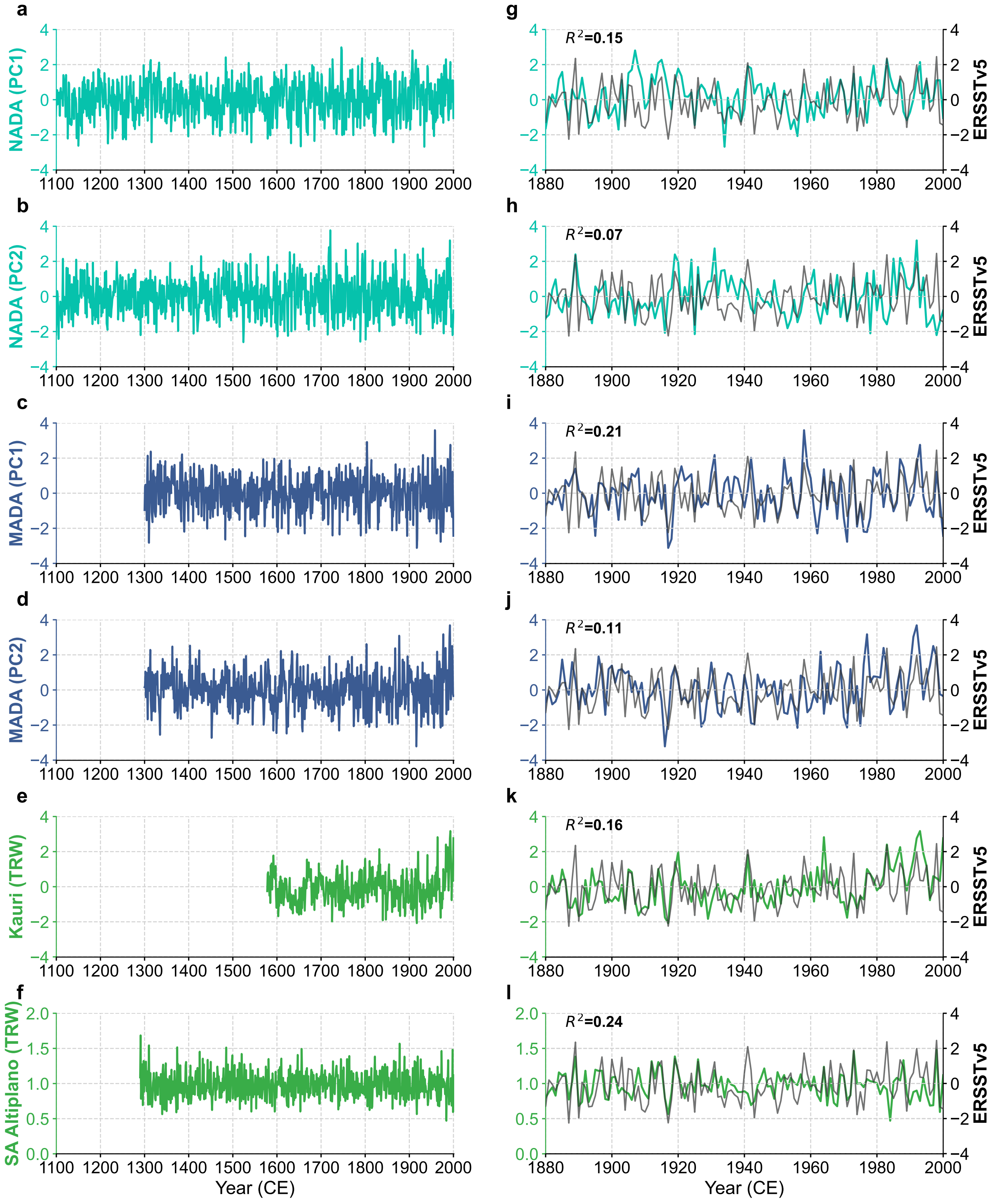

Supplementary Fig. 13. The six best ENSO predictors from Li et al.[6] against the the December-February (DJF) seasonally averaged Extended Reconstructed Sea Surface Temperature v5 (ERSSTv5)[4]. (a-f) Timeseries of the six best predictors from Li et al.[6], including the first two princile components of NADA and MADA, the Kauri tree-ring composite[12], as well as the South American Altiplano tree-ring composite[13], over the past millennium (1100-2000 CE). **(g-l)** Same as in (a-f), but over the instrumental period (1881-2000 CE). Validation is performed against the December-February (DJF) seasonally averaged Niño 3.4 calculated from Extended Reconstructed Sea Surface Temperature, Version 5 (ERSSTv5)[4] over the instrumental period (1881-2000 CE). R^2=coefficient of determination.

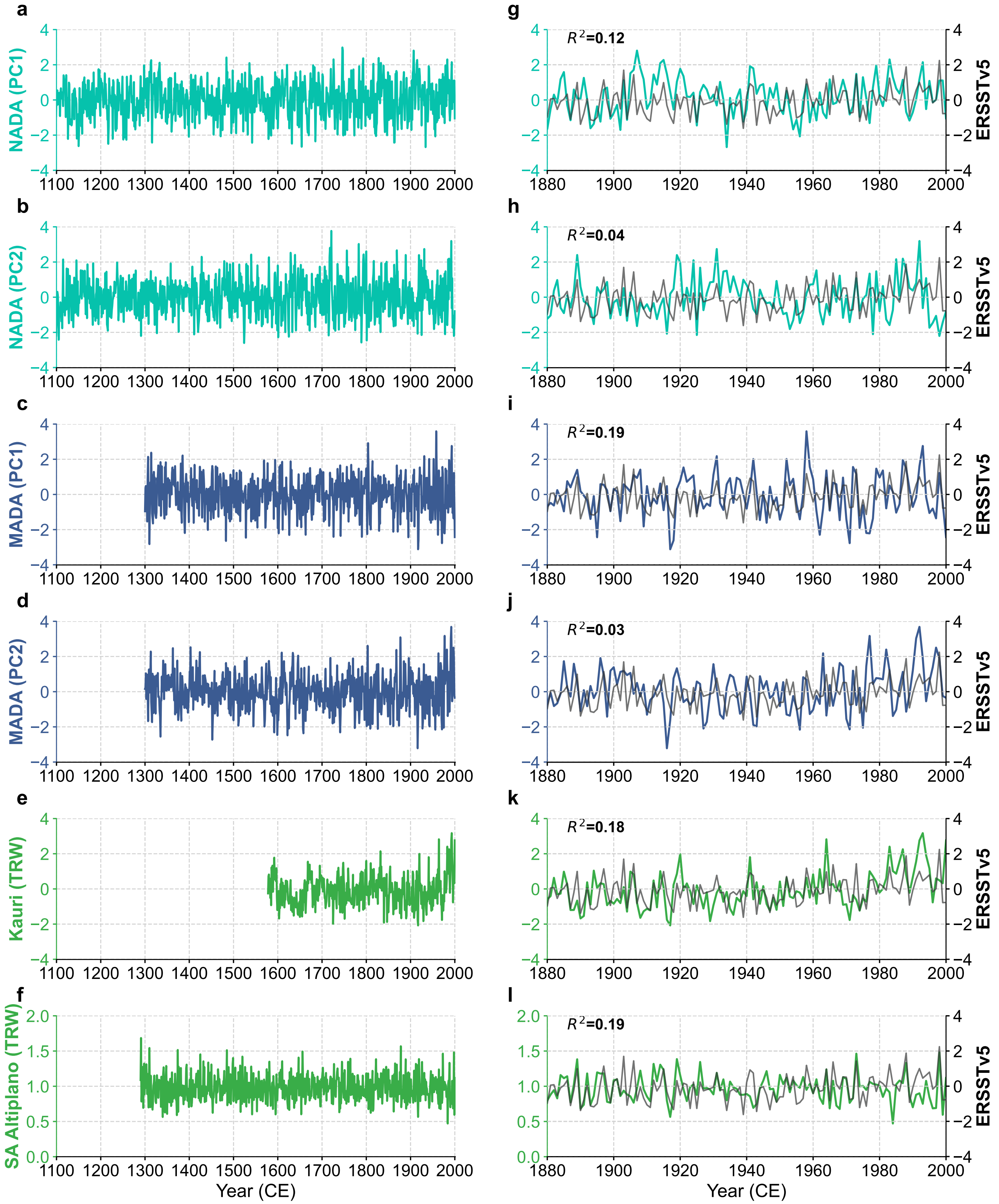

Supplementary Fig. 14. The six best ENSO predictors from Li et al.[6] against the the July-September (JAS) seasonally averaged Extended Reconstructed Sea Surface Temperature v5 (ERSSTv5)[4]. (a-f) Timeseries of the six best predictors from Li et al.[6], including the first two princile components of NADA and MADA, the Kauri tree-ring composite[12], as well as the South American Altiplano tree-ring composite[13], over the past millennium (1100-2000 CE). **(g-l)** Same as in (a-f), but over the instrumental period (1881-2000 CE). Validation is performed against the December-February (DJF) seasonally averaged Niño 3.4 calculated from Extended Reconstructed Sea Surface Temperature, Version 5 (ERSSTv5)[4] over the instrumental period (1881-2000 CE). R^2=coefficient of determination.

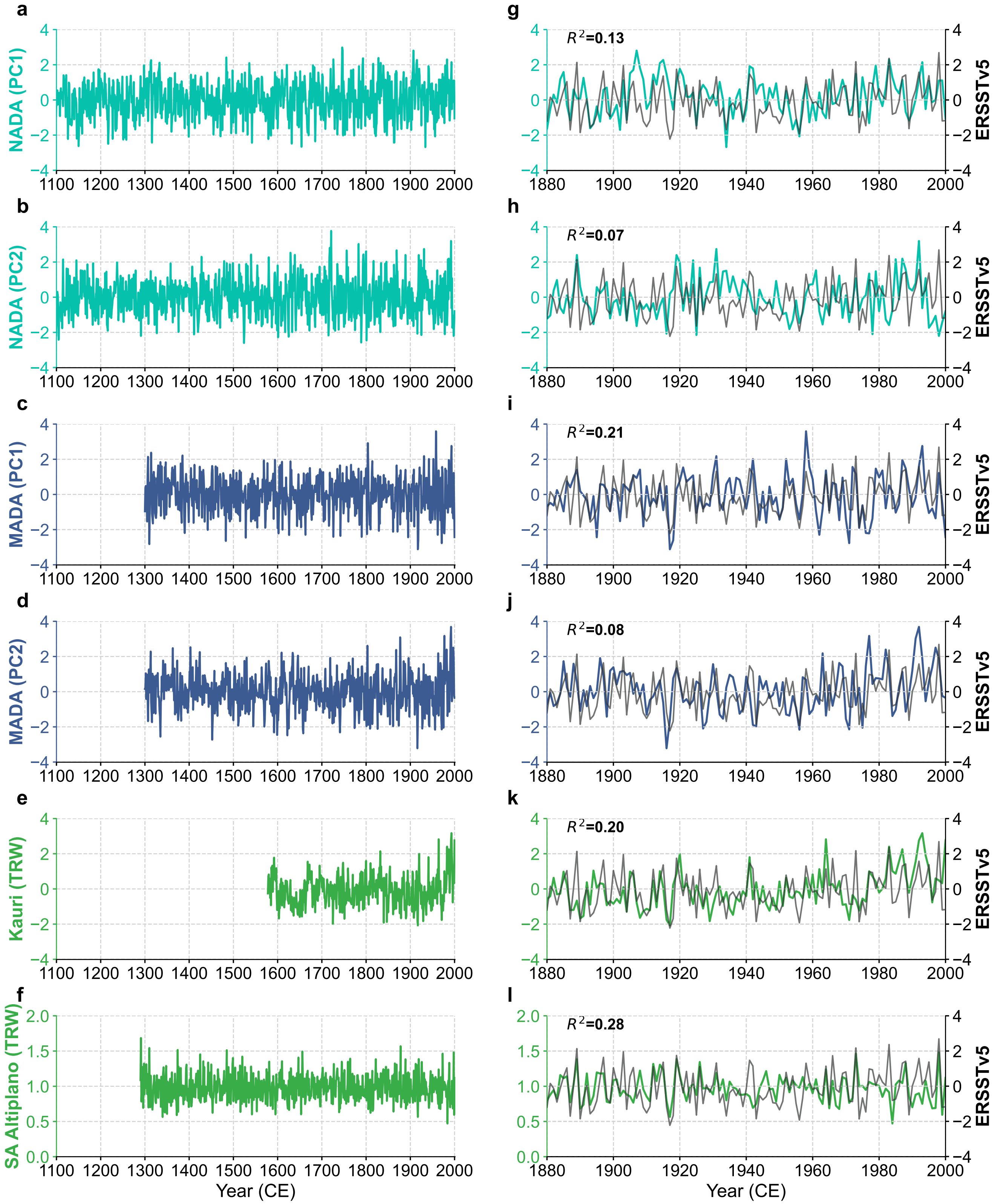

Supplementary Fig. 15. The six best ENSO predictors from Li et al.[6] against the the October-December (OND) seasonally averaged Extended Reconstructed Sea Surface Temperature v5 (ERSSTv5)[4]. (a-f) Timeseries of the six best predictors from Li et al.[6], including the first two princile components of NADA and MADA, the Kauri tree-ring composite[12], as well as the South American Altiplano tree-ring composite[13], over the past millennium (1100-2000 CE). **(g-l)** Same as in (a-f), but over the instrumental period (1881-2000 CE). Validation is performed against the December-February (DJF) seasonally averaged Niño 3.4 calculated from Extended Reconstructed Sea Surface Temperature, Version 5 (ERSSTv5)[4] over the instrumental period (1881-2000 CE). R^2=coefficient of determination.

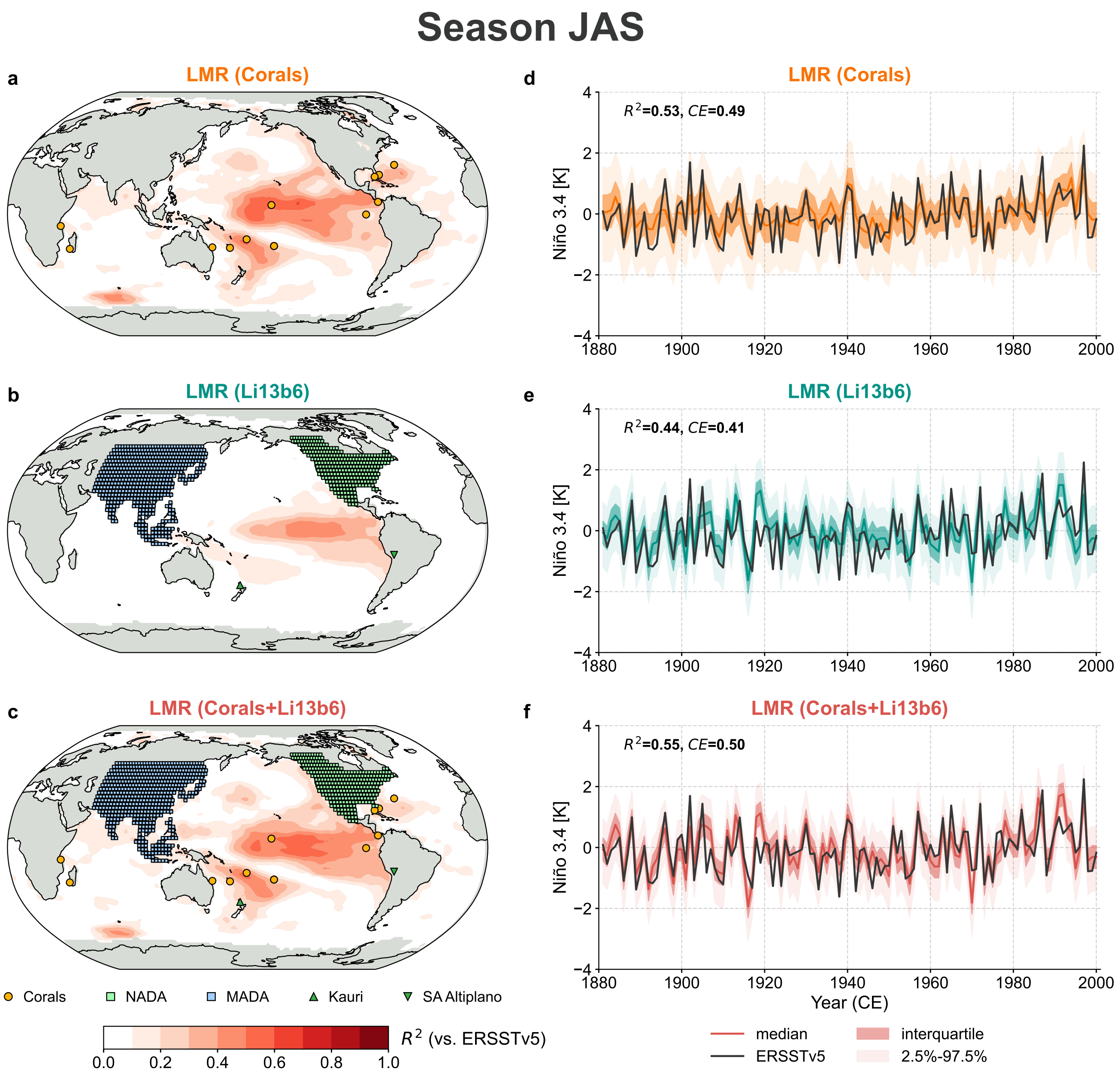

Supplementary Fig. 16. Validation of the Last Millennium Reanalysis (LMR)[1,2] July-September (JAS) sea surface temperature reconstructions over the instrumental period (1881-2000 CE). The labels LMR (Corals), LMR (Li13b6), and LMR (Corals+Li13b6) denote the reconstructions assimilating corals reaching back before 1750 CE from the Ocean2k compilation[8] updated with the latest Palmyra data[9], the six best predictors from Li et al.[6] (denoted as Li13b6), and both data sources, respectively. **(a-c)** Spatial verification of the median field of the reconstructed July-September (JAS) surface temperature. Validation is performed against the Extended Reconstructed Sea Surface Temperature, Version 5 (ERSSTv5)[4]. The orange dots denote the location of the corals, the mint and blue squares denote the location of the North American Drought Atlas (NADA) (Version 2a)[10] and Monsoon Asia Drought Atlas (MADA)[11] sites, the green upward triangle denotes the location of the Kauri tree-ring composite[12], and the green downward triangle denotes the location of the South America Altiplano (SA Altiplano) tree-ring composite[13]. **(d-f)** Temporal verification of the median of the LMR reconstructed JAS Niño 3.4 series (colored curves) against the ERSSTv5 derived Niño 3.4 (black solid curve). For each reconstruction, dark shading denotes the interquartile range, and light shading denotes the central 95% region, from 2.5% to 97.5%. R^2=coefficient of determination, CE=coefficient of efficiency[3].

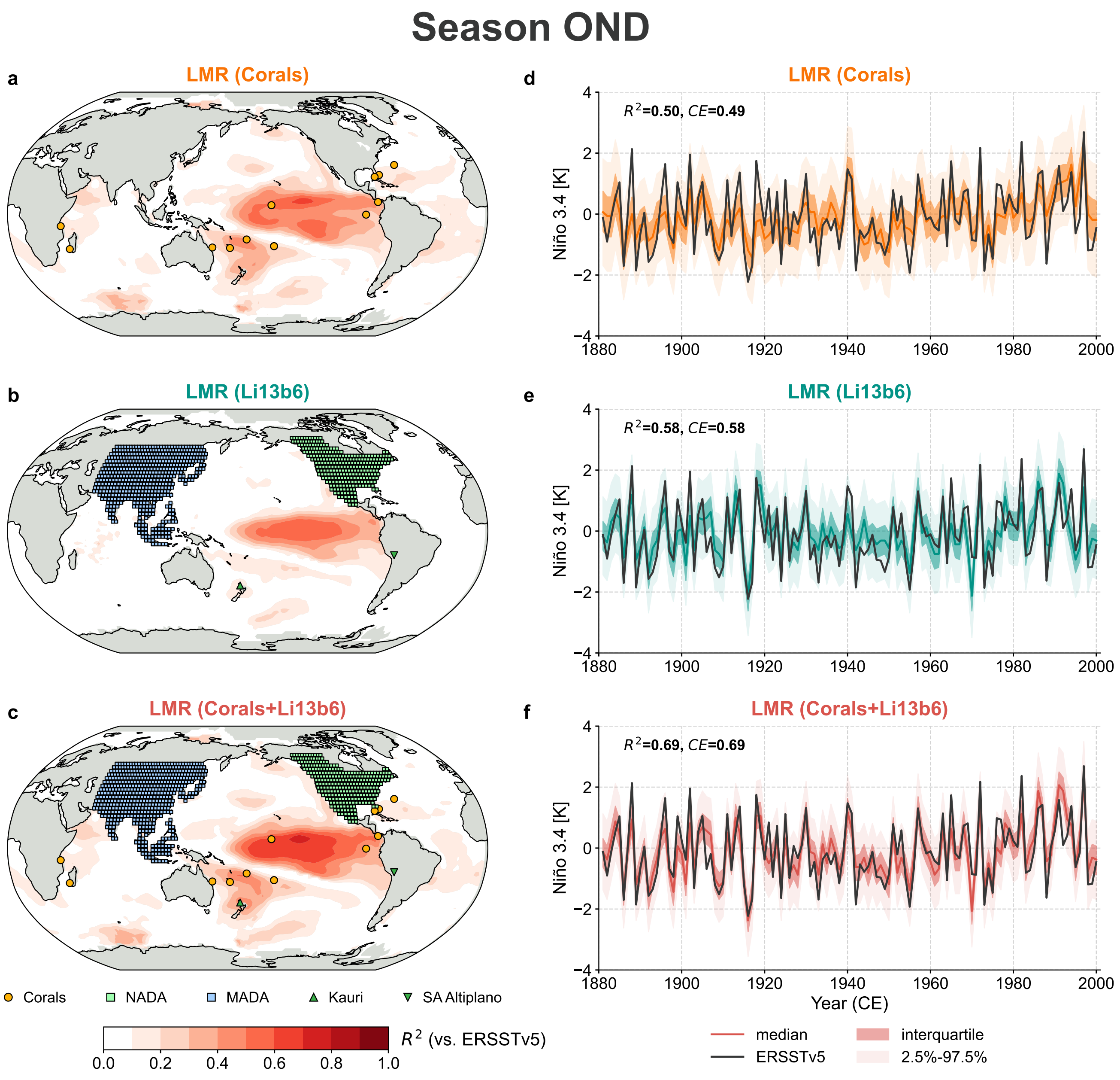

Supplementary Fig. 17. Validation of the Last Millennium Reanalysis (LMR)[1,2] October-December (OND) sea surface temperature reconstructions over the instrumental period (1881-2000 CE). The labels LMR (Corals), LMR (Li13b6), and LMR (Corals+Li13b6) denote the reconstructions assimilating corals reaching back before 1750 CE from the Ocean2k compilation[8] updated with the latest Palmyra data[9], the six best predictors from Li et al.[6] (denoted as Li13b6), and both data sources, respectively. **(a-c)** Spatial verification of the median field of the reconstructed October-December (OND) surface temperature. Validation is performed against the Extended Reconstructed Sea Surface Temperature, Version 5 (ERSSTv5)[4]. The orange dots denote the location of the corals, the mint and blue squares denote the location of the North American Drought Atlas (NADA) (Version 2a)[10] and Monsoon Asia Drought Atlas (MADA)[11] sites, the green upward triangle denotes the location of the Kauri tree-ring composite[12], and the green downward triangle denotes the location of the South America Altiplano (SA Altiplano) tree-ring composite[13]. **(d-f)** Temporal verification of the median of the LMR reconstructed OND Niño 3.4 series (colored curves) against the ERSSTv5 derived Niño 3.4 (black solid curve). For each reconstruction, dark shading denotes the interquartile range, and light shading denotes the central 95% region, from 2.5% to 97.5%. R^2=coefficient of determination, CE=coefficient of efficiency[3].

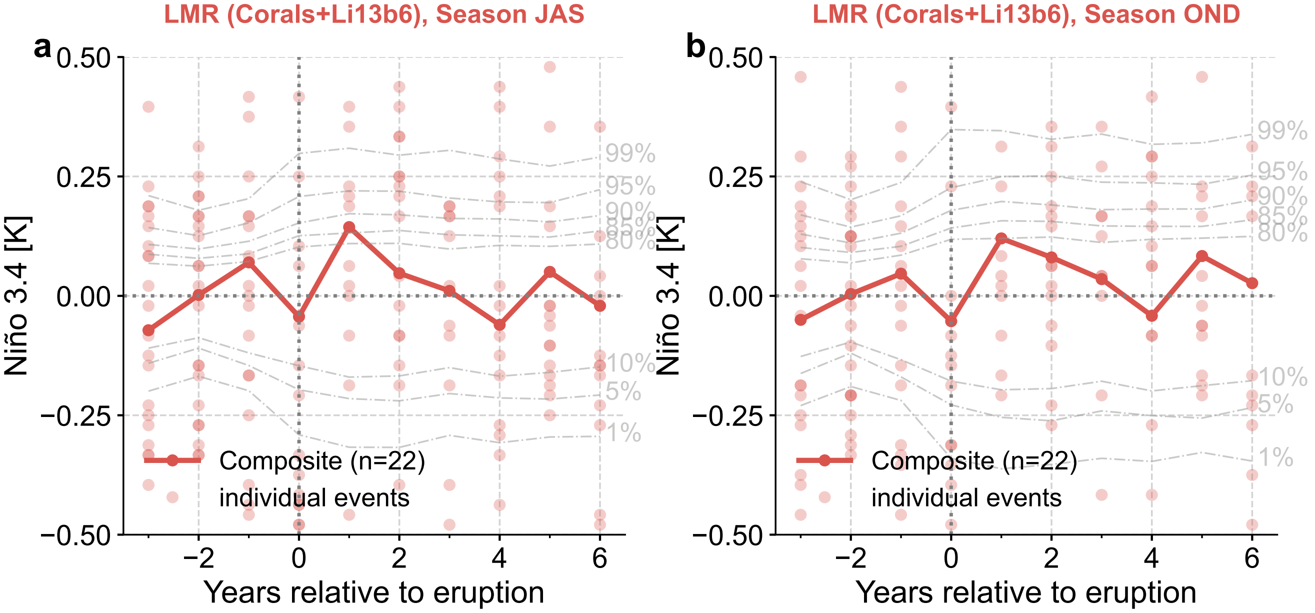

Supplementary Fig. 18. A superposed epoch analysis (SEA) of the Last Millennium Reanalysis (LMR)[1,2] July-September (JAS) and October-December (OND) Niño 3.4 reconstructions. The reconstruction is denoted as LMR (Corals+Li13b6), assimilating both corals and the six best predictors from Li et al.[6]. The SEA is regarding all the 22 events over the past millennium.

Supplementary Text 5: A comparison of historical SST analyses

In our reconstructions, a spatially completed version of HadCRUT4.6[29] leveraging the GraphEM[30] algorithm is used as the target for calibration, and the Extended Reconstructed Sea Surface Temperature v5 (ERSSTv5)[4] is used for validation. We compare them along with the NASA Goddard Institute for Space Studies (GISS) Surface Temperature Analysis (GISTEMP)[31], which is used as the default calibration target in the official LMR products[1,2]. Supplementary Fig. 19 shows that the long term trend of the DJF Niño 3.4 signal in the three products are slightly different, while the high-frequency signal is very consistent. The fact that we use different targets for calibration and validation and still obtain reconstructions with remarkably high skill suggests that reconstructions are robust to these differences.

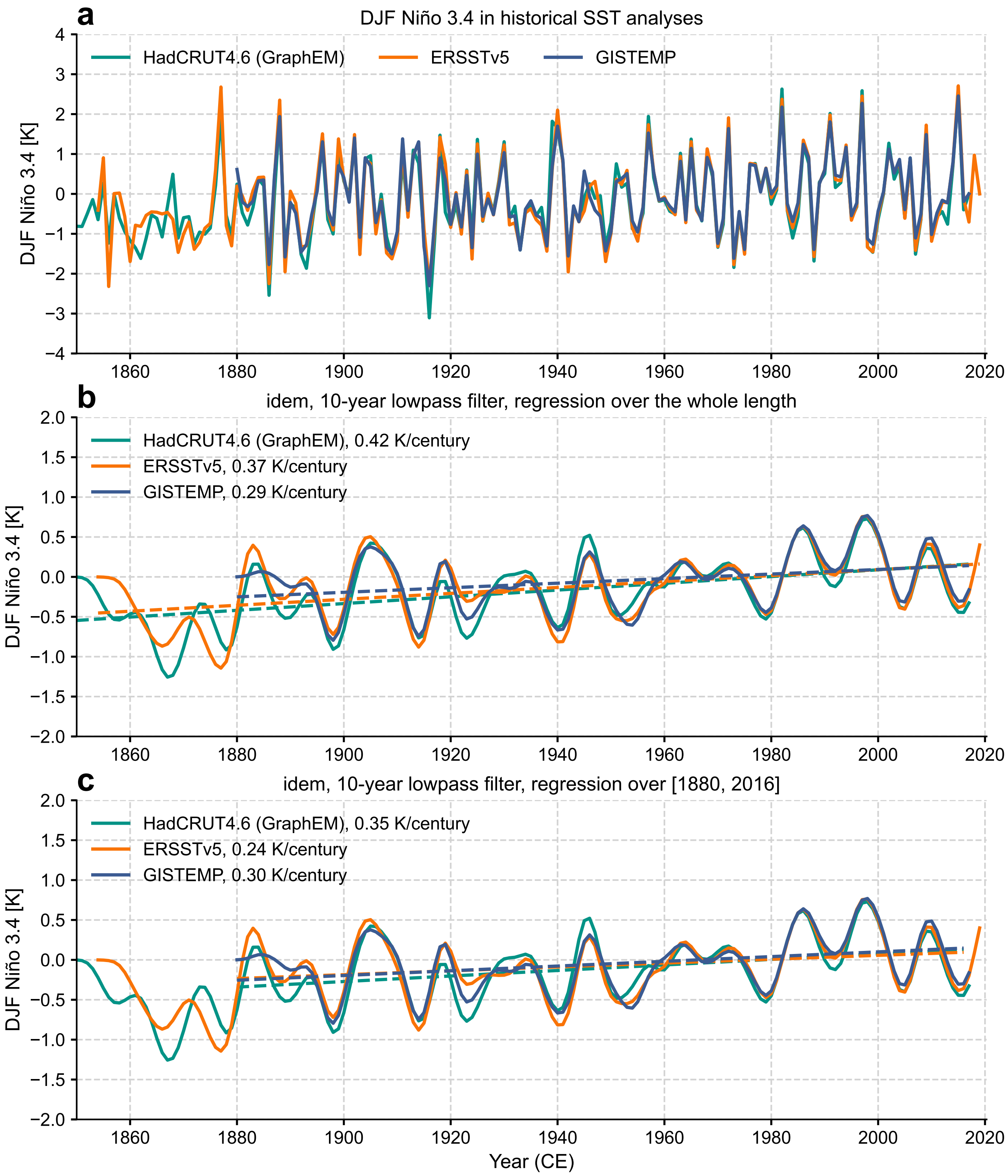

Supplementary Fig. 19. Historical SST analyses products of December-February (DJF) Niño 3.4. The products include a spatially completed version of HadCRUT4.6[29] leveraging the GraphEM[30] algorithm, the Extended Reconstructed Sea Surface Temperature v5 (ERSSTv5)[4], and the NASA Goddard Institute for Space Studies (GISS) Surface Temperature Analysis (GISTEMP)[31].

Supplementary Text 6: Investigating nonstationarity

We conduct a list of pseudoproxy experiments (PPEs) to investigate the nonstationarity we observe in our reconstruction and the Li13 reconstruction (Supplementary Fig. 20), and the results are presented in Supplementary Fig. 21.

In the PPEs, we utilize the isotope-enabled CESM simulation (iCESM)[32,33] as our "nature run" (the simulated truth), since it is expected to exhibit a strong climate response to volcanism[32,33]. We then generate several versions of pseudoproxies following a simple linear relationship $p_k = T_k + w_k$, where p_k denotes the k-th pseudoproxy value, T_k the iCESM simulated surface temperature at the gridcell nearest to the target proxy, and w_k the white noise with certain signal-to-noise ratio (SNR). The experimental design includes:

(i) nearly noiseless pseudoproxies ($SNR = 100$), with realistic spatial availability but constant temporal availability (no gaps);

(ii) same as (i), but with realistic temporal availability;

(iii) same as (ii), but with $SNR = 1$;

(iv) same as (iii), but the pseudoproxies are temporally perturbed over the pre-instrumental era using a probabilistic model of age errors in layer-counted chronologies (BAM)[34];

(v) Same as (iv), but the pseudoproxies are generated from another realization of the BAM perturbation.

Comparing the result of (ii) against that of (i) (Supplementary Fig. 21b,g against Supplementary Fig. 21a,f), we see that the realistic temporal availability alone is not a major cause of the non-stationarity we observe in the reconstructions. The reason is that the Palmyra coral record is located near the edge of the Niño 3.4 region, and the tree-ring based predictors are treated as ENSO proxies and are put at the center of the Niño 3.4 region. Both sources are at least annually resolved, so the essential composition of the assimilated proxy database is not as temporally heterogeneous as the whole database, hence not a severe issue.

Comparing the result of (iii) against that of (ii) (Supplementary Fig. 21c,h against Supplementary Fig. 21b,g), we see that the additional proxy noise with SNR=1 has little impact on the non-stationarity, but can help reduce the significance level of the year 1 response by around 5%. Yet, we note that the exact reduction value is a variable dependent on the SNR in practice.

Comparing the result of (iv) against that of (iii) (Supplementary Fig. 21d,i against Supplementary Fig. 21c,h), we see that even minor age uncertainties can create similar non-stationarity as observed in our reconstructions while keeping a similar SEA significance level, even with SNR=1. However, in other cases such as another realization of the BAM perturbation, the year 1 ENSO response vanishes (Supplementary Fig. 21e,j).

In addition, we test the impact of non-stationarity by stabilizing our reconstruction LMR (Corals+Li13b6). We inflate the timeseries by 1.5 times over the timespan 1100-1850 CE so as to achieve a more heterogeneous variability across the whole timespan 1100-2000 CE (Supplementary Fig. 22b), yet we see that the significance level of the post-eruption responses in SEA is essentially not affected (Supplementary Fig. 22c against Supplementary Fig. 22d).

We therefore would argue that the observed non-stationarity in our reconstruction can be a combination effect of multiple factors such as proxy error and proxy dating uncertainty, yet its impact on the post-eruption ENSO responses is not systematic, and it is unlikely to be the leading cause of weak post-eruption ENSO responses in our reconstructions.

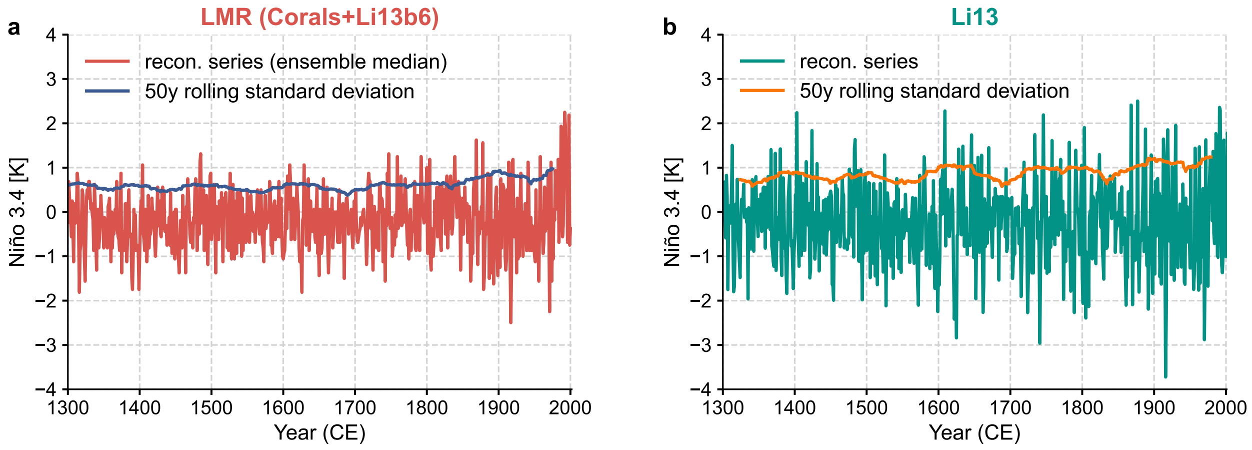

Supplementary Fig. 20. Nonstationarity in the Last Millennium Reanalysis (LMR)[1,2] reconstruction and the Li et al. reconstruction[6]. (a) The ensemble median series of LMR (Corals+Li13b6) along with its 50-year rolling standard deviation. **(b)** The Li et al.[6] reconstruction (denoted as Li13) along with its 50-year rolling standard deviation.

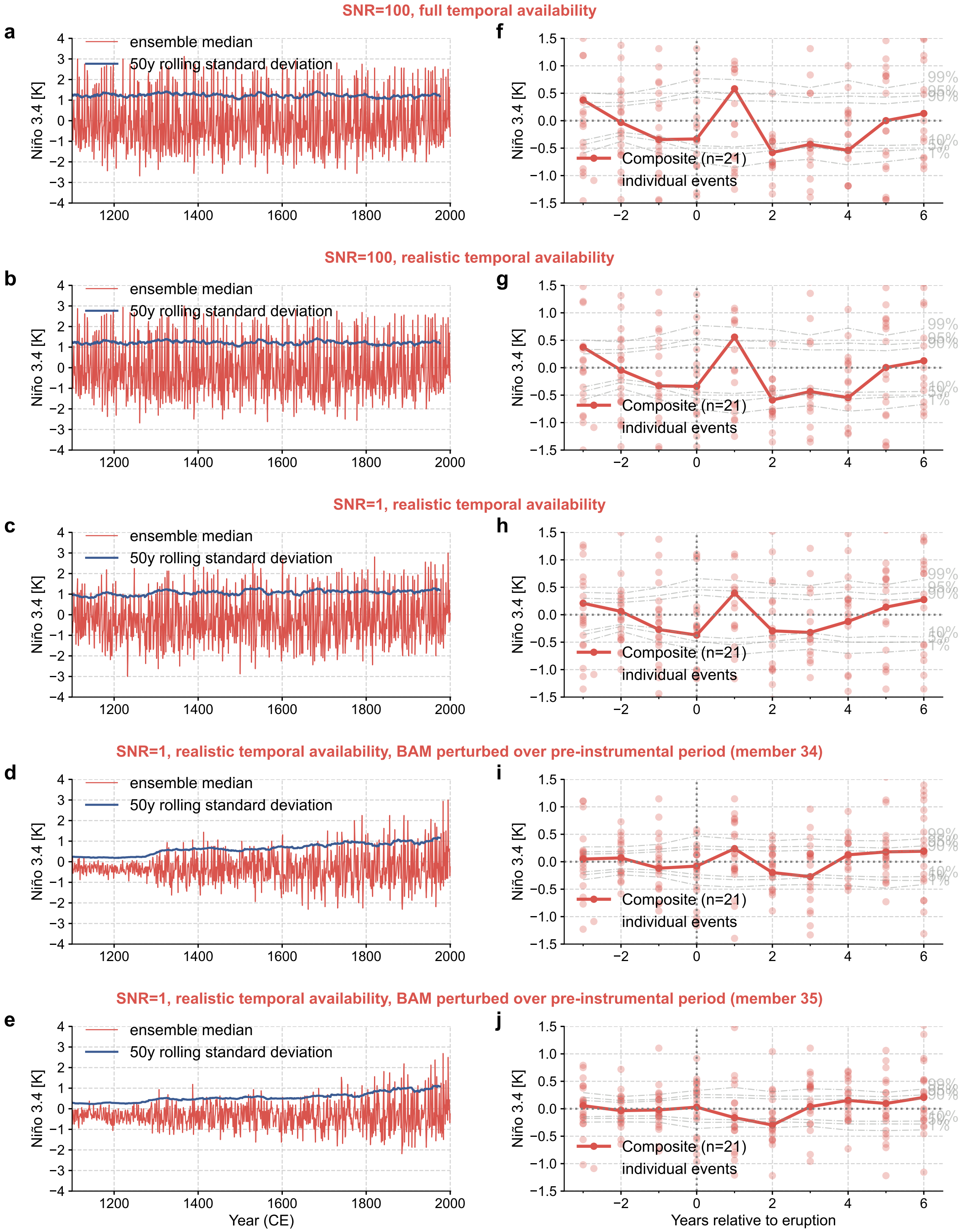

Supplementary Fig. 21. Analysis of the pseudoproxy experiments (PPEs). The isotope-enabled CESM simulation (iCESM)[32,33] is used as the nature run of the PPEs. The left column shows the ensemble median series along with the 50-year rolling standard deviation of each reconstruction assimilating different pseudoproxies, and the right column shows the corresponding SEA regarding 21 large eruptions (1167, 1176, 1195, 1227, 1258, 1275, 1284, 1341, 1452, 1459, 1584, 1600, 1641, 1693, 1719, 1783, 1809, 1815, 1835, 1883, 1991) selected based on Gao et al.[21]. **(a, f)** The case in which the pseudoproxies are almost noiseless, with the signal-to-noise ratio (SNR) equals to 100, and the pseudoproxies have full annual temporal availability over 1100-2000 CE. **(b, g)** Same as (a, f) but the pseudoproxies have realistic temporal availability. **(c, h)** Same as (b, g), but the pseudoproxies have SNR=1. **(d, i)** Same as (c, h), but he pseudoproxies are temporally perturbed over pre-instrumental period using a probabilistic model of age errors in layer-counted chronologies (BAM)[34]. **(e, j)** Same as (d, i), but the pseudoproxies are generated from another ensemble member of BAM.

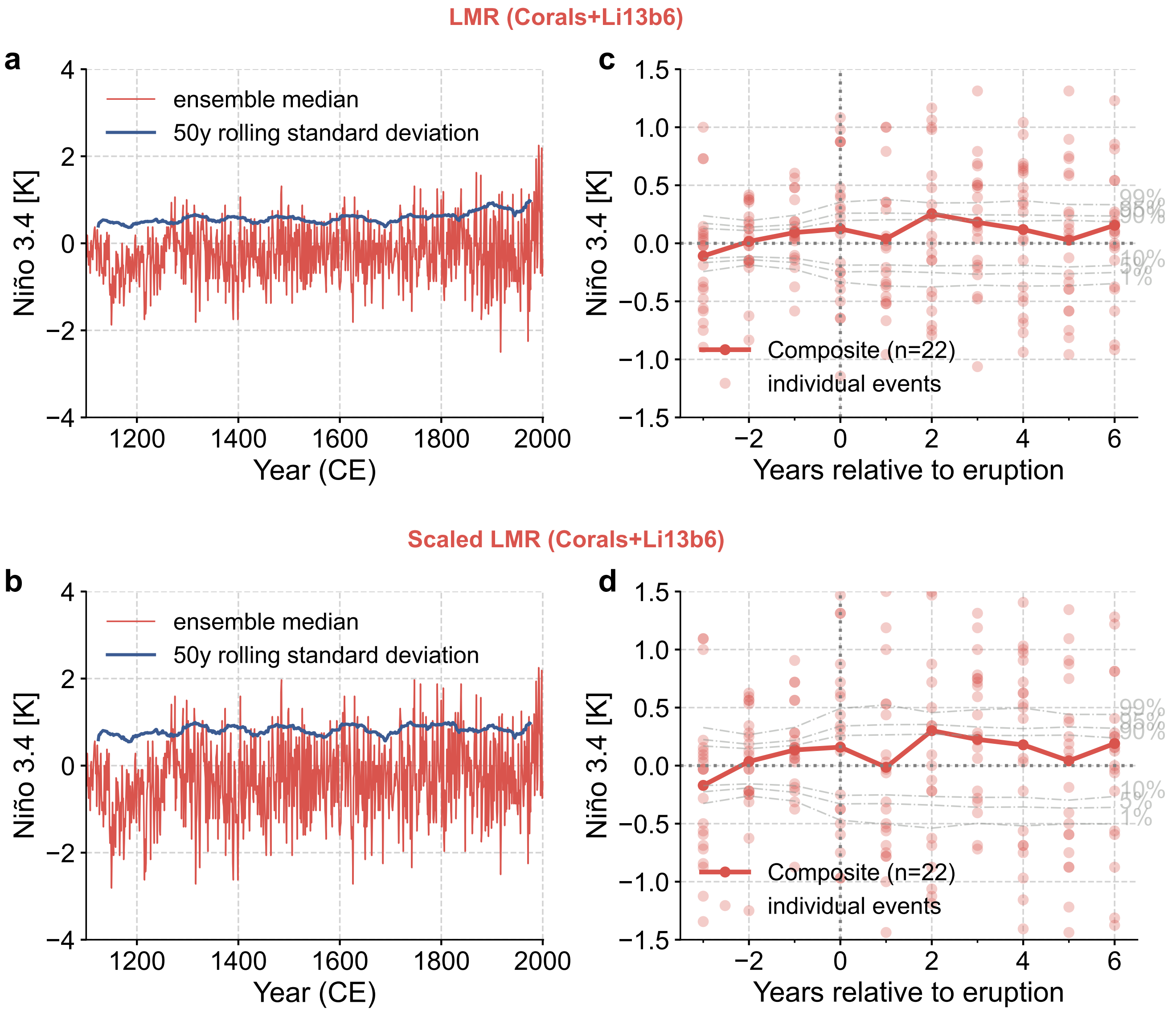

Supplementary Fig. 22. A test of the impact of scaling the Last Millennium Reanalysis (LMR)[1,2] reconstructed Niño 3.4 series on superposed epoch analysis (SEA). (a,b) The ensemble median series of LMR (Corals+Li13b6) along with its scaled series. The amplitude of the scaled series is scaled by 1.5 times of that of the original series over the timespan 1100-1850 CE so as to alleviate the non-stationarity. **(c,d)** The SEA on LMR (Coral+Li13b6) and its scaled series regarding the large eruption events selected based on VSS>6 over the timespan 1100-1900 CE.

Supplementary Text 7: Exploring discrepancies

A recent review[35] argued that most current-era GCMs, as well as a majority of published reconstructions, agree on a significant ENSO response to volcanism (year 0 or 1). Yet our analysis finds a much weaker connection, and one that tends to emerge only in year 1 or 2 following an eruption. One possibility is, again, that the study used a different selection of events. We thus repeat our analysis on the most recent set of reconstructions used by these authors, as well as the Paleo Hydrodynamics Data Assimilation product (PHYDA)[7] (Supplementary Fig. 23), using the "Agreed Event Years" from their Table 12.2. The result is shown in Supplementary Fig. 24, suggesting that none of the reconstructions show significant post-eruption ENSO responses. In addition, we applied the SEA using the event years based on our criterion used in the main text (VSSI>6), and the same conclusion is obtained as shown in Supplementary Fig. 25. Note that these reconstructions are not independent: many use a number of common predictors, and one (McGregor et al.[17], M10 hereafter) is itself a composite of several previous reconstructions, potentially counting common predictors multiple times.

The lack of a robust connection between explosive volcanism and our reconstructed Niño 3.4 index is also at odds with results from climate model simulations of the past millennium, some of which show a significant year 1 response (Supplementary Fig. 7,8, Supplementary Text 3), particularly when using relative sea surface temperature (RSST)[24] (Supplementary Fig. 9,10). This behavior, however, is not universal in this set of models: even with the RSST conversion, SEA still produces inconsistent ENSO responses (see Supplementary Text 3 for details), as pointed out in D20. It remains to be seen whether analyses of the more recent PMIP4 "past2k" simulations, which employ the eVolv2k forcing[23], display more coherent responses.

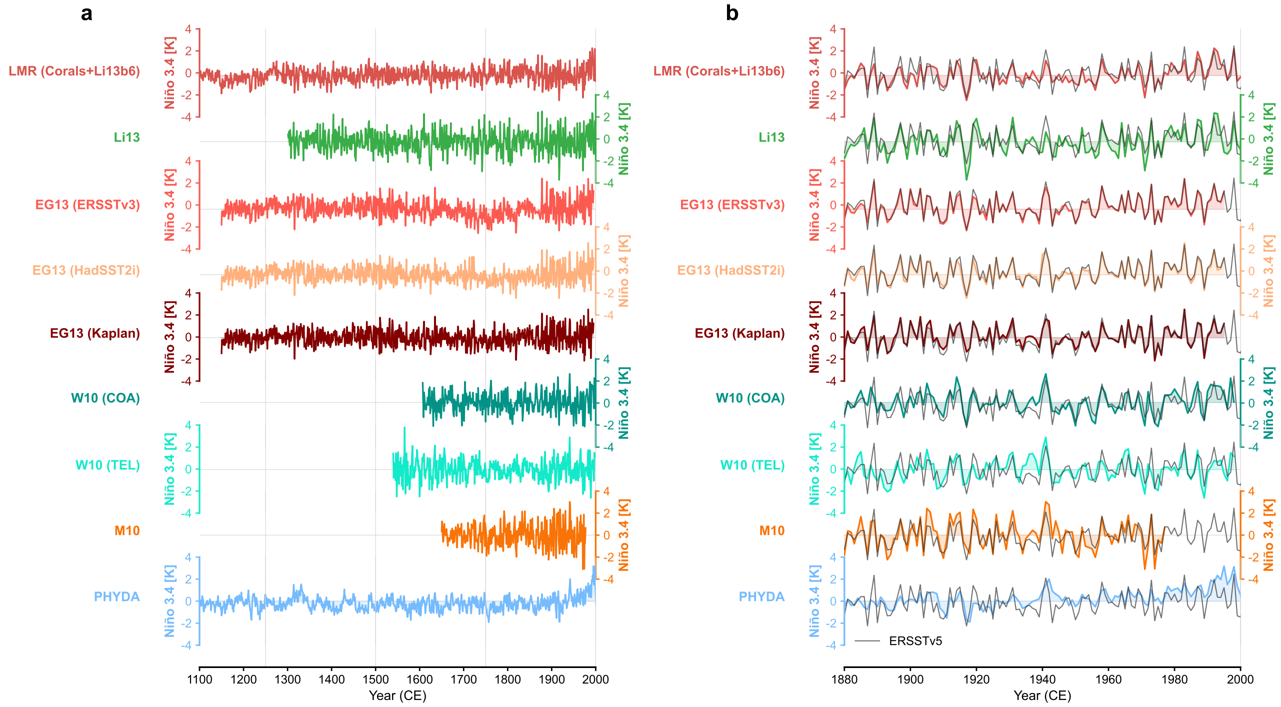

Supplementary Fig. 23. A showcase of the Last Millennium Reanalysis (LMR)[1,2] reconstruction along with previous ENSO reconstructions. (a) The timeseries over 1100-2000 CE. The previous ENSO reconstructions include Li et al. (Li13)[6], Emile-Geay et al. (EG13)[36], Wilson et al. (W10)[37], McGregor et al. (M10)[17], and PHYDA[7]. **(b)** Same as (a) but over the 1880-2000 CE period, along with the ERSSTv5 (Extended Reconstructed Sea Surface Temperature v5) instrumental estimate of Niño 3.4[4].

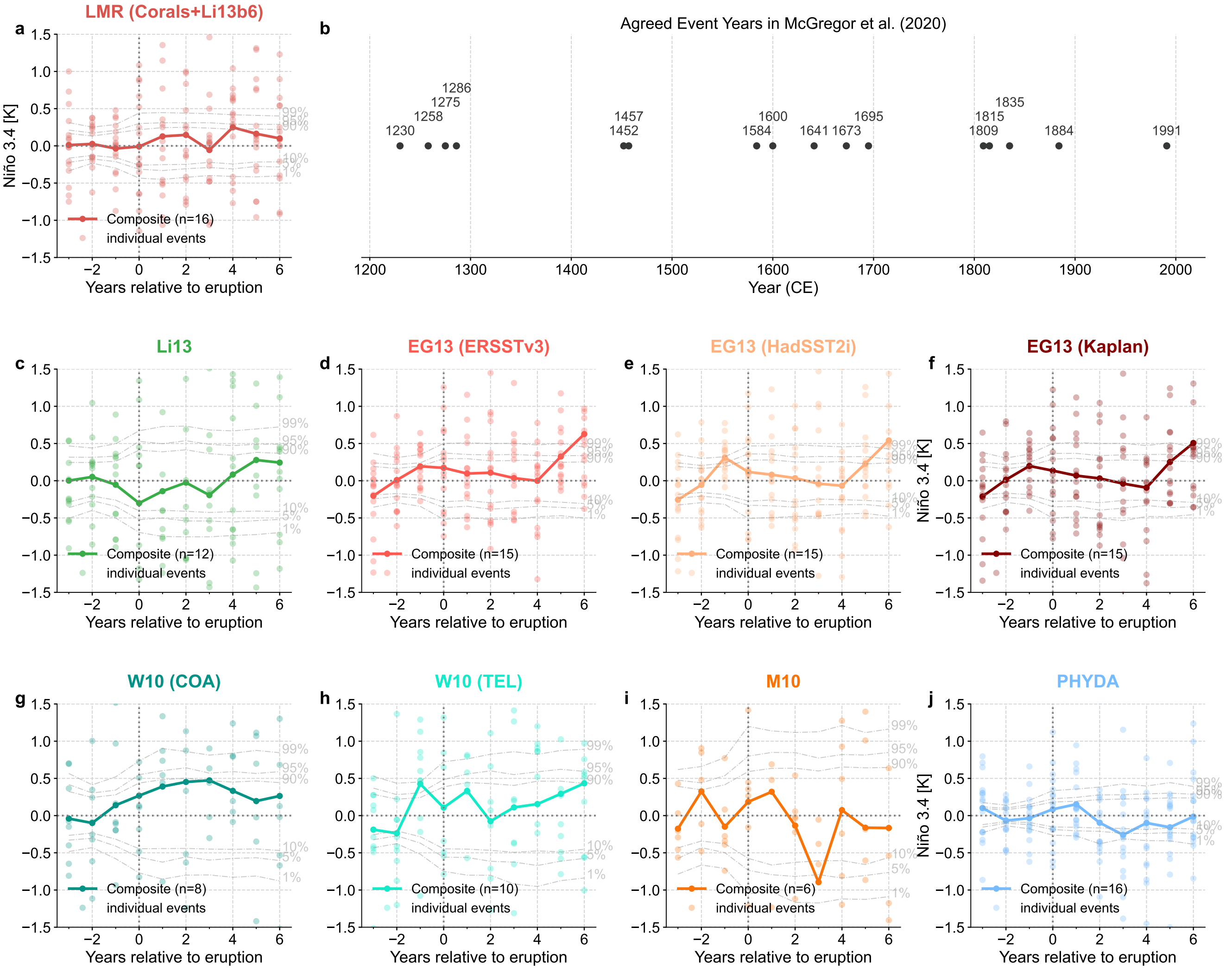

Supplementary Fig. 24. Superposed epoch analysis (SEA) of the ENSO reconstructions in Supplementary Fig. 23. (a, c-i) SEA of the reconstructions regarding the event years listed in (b) covered by the timespan of each reconstruction. **(b)** The agreed event years listed in Table 12.2 of McGregor et al.[35].

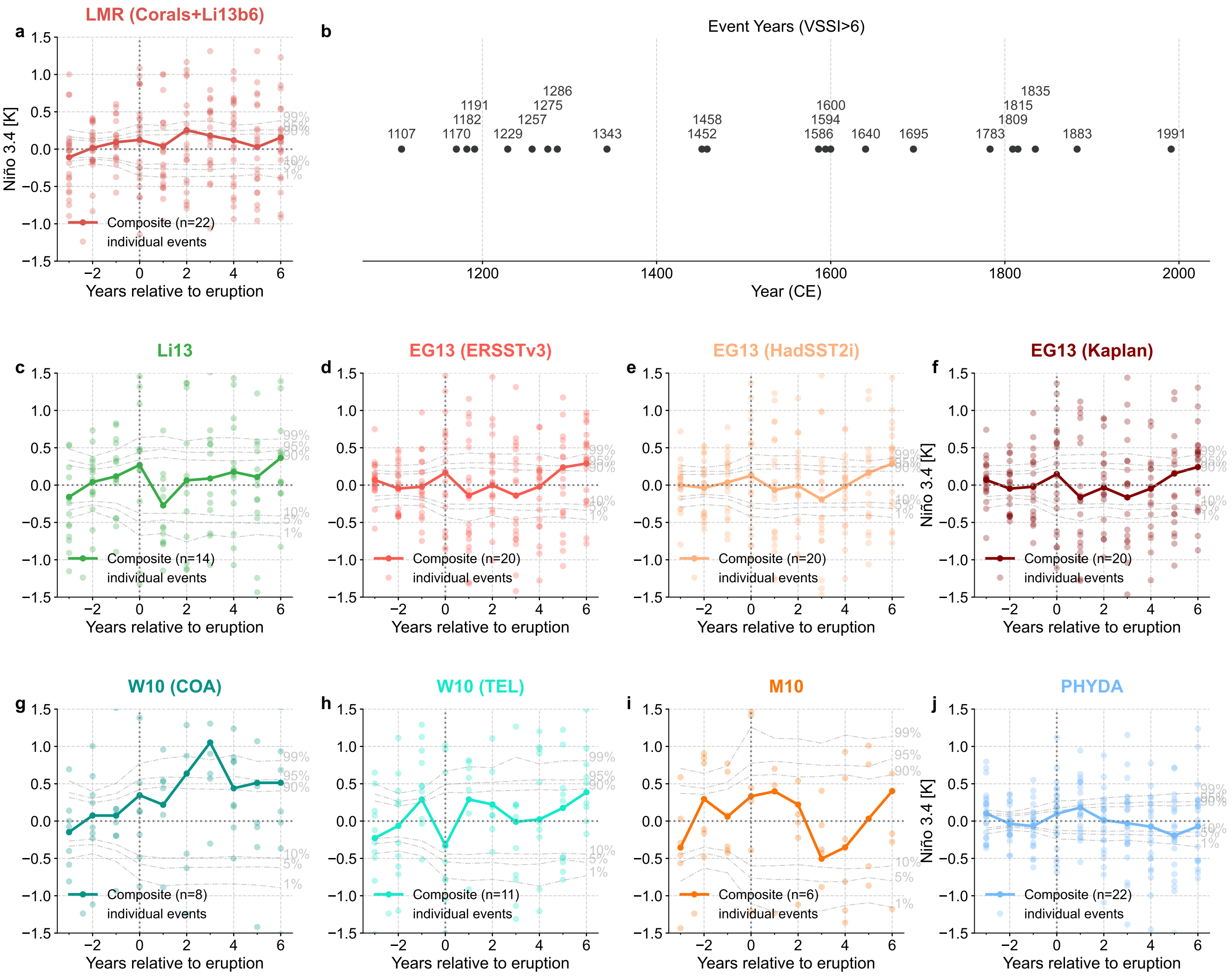

Supplementary Fig. 25. Superposed epoch analysis (SEA) of the ENSO reconstructions in Supplementary Fig. 23. (a, c-i) SEA of the reconstructions regarding the event years listed in (b) covered by the timespan of each reconstruction. **(b)** The event years based on the volcanic stratospheric sulfur injection (VSSI) greater than 6 according to eVolv2k version 3[23] and Toohey et al.[38].

References

1. Hakim, G. J. *et al.* The last millennium climate reanalysis project: Framework and first results. *J. Geophys. Res. Atmospheres* **121**, 2016JD024751, DOI: 10.1002/2016JD024751 (2016).
2. Tardif, R. *et al.* Last Millennium Reanalysis with an expanded proxy database and seasonal proxy modeling. *Clim. Past* **15**, 1251–1273, DOI: https://doi.org/10.5194/cp-15-1251-2019 (2019).
3. Nash, J. & Sutcliffe, J. River flow forecasting through conceptual models part I – A discussion of principles. *J. Hydrol.* **10**, 282–290, DOI: 10.1016/0022-1694(70)90255-6 (1970).
4. Huang, B. *et al.* Extended Reconstructed Sea Surface Temperature, Version 5 (ERSSTv5): Upgrades, Validations, and Intercomparisons. *J. Clim.* **30**, 8179–8205, DOI: 10.1175/JCLI-D-16-0836.1 (2017).
5. Bunge, L. & Clarke, A. J. A Verified Estimation of the El Niño Index Niño-3.4 since 1877. *J. Clim.* **22**, 3979–3992, DOI: 10.1175/2009JCLI2724.1 (2009).
6. Li, J. *et al.* El Niño modulations over the past seven centuries. *Nat. Clim. Chang.* **3**, 822–826, DOI: 10.1038/nclimate1936 (2013).
7. Steiger, N. J., Smerdon, J. E., Cook, E. R. & Cook, B. I. A reconstruction of global hydroclimate and dynamical variables over the Common Era. *Sci. Data* **5**, 1–15, DOI: 10.1038/sdata.2018.86 (2018).
8. Tierney, J. E. *et al.* Tropical sea surface temperatures for the past four centuries reconstructed from coral archives. *Paleoceanography* **30**, 226–252, DOI: 10.1002/2014PA002717 (2015).
9. Dee, S. G. *et al.* No consistent ENSO response to volcanic forcing over the last millennium. *Science* **367**, 1477–1481, DOI: 10.1126/science.aax2000 (2020).
10. Cook, E. R., Woodhouse, C. A., Eakin, C. M., Meko, D. M. & Stahle, D. W. Long-Term Aridity Changes in the Western United States. *Science* **306**, 1015–1018, DOI: 10.1126/science.1102586 (2004).
11. Cook, E. R. *et al.* Asian Monsoon Failure and Megadrought During the Last Millennium. *Science* **328**, 486–489, DOI: 10.1126/science.1185188 (2010).
12. Fowler, A. M., Boswijk, G., Gergis, J. & Lorrey, A. ENSO history recorded in Agathis australis (kauri) tree rings. Part A: kauri's potential as an ENSO proxy. *Int. J. Climatol.* **28**, 1–20, DOI: 10.1002/joc.1525 (2008).
13. Morales, M. S. *et al.* Precipitation changes in the South American Altiplano since 1300 AD reconstructed by tree-rings. *Clim. Past* **8**, 653–666, DOI: https://doi.org/10.5194/cp-8-653-2012 (2012).
14. Haurwitz, M. W. & Brier, G. W. A Critique of the Superposed Epoch Analysis Method: Its Application to SolarWeather Relations. *Mon. Weather. Rev.* **109**, 2074–2079, DOI: 10.1175/1520-0493(1981)109<2074:ACOTSE>2.0.CO;2 (1981).
15. Delworth, T. L. *et al.* GFDL's CM2 Global Coupled Climate Models. Part I: Formulation and Simulation Characteristics. *J. Clim.* **19**, 643–674, DOI: 10.1175/JCLI3629.1 (2006).
16. Wittenberg, A. T., Rosati, A., Lau, N.-C. & Ploshay, J. J. GFDL's CM2 Global Coupled Climate Models. Part III: Tropical Pacific Climate and ENSO. *J. Clim.* **19**, 698–722, DOI: 10.1175/JCLI3631.1 (2006).
17. McGregor, S., Timmermann, A. & Timm, O. A unified proxy for ENSO and PDO variability since 1650. *Clim. Past* **6**, 1–17, DOI: https://doi.org/10.5194/cp-6-1-2010 (2010).
18. Stevenson, S., Otto-Bliesner, B., Fasullo, J. & Brady, E. El Niño Like Hydroclimate Responses to Last Millennium Volcanic Eruptions. *J. Clim.* **29**, 2907–2921, DOI: 10.1175/JCLI-D-15-0239.1 (2016).
19. Braconnot, P. *et al.* Evaluation of climate models using palaeoclimatic data. *Nat. Clim. Chang.* **2**, 417–424, DOI: 10.1038/nclimate1456 (2012).
20. Otto-Bliesner, B. L. *et al.* Climate Variability and Change since 850 CE: An Ensemble Approach with the Community Earth System Model. *Bull. Am. Meteorol. Soc.* **97**, 735–754, DOI: 10.1175/BAMS-D-14-00233.1 (2015).
21. Gao, C., Robock, A. & Ammann, C. Volcanic forcing of climate over the past 1500 years: An improved ice core-based index for climate models. *J. Geophys. Res. Atmospheres* **113**, DOI: 10.1029/2008JD010239 (2008).
22. Crowley, T. J. *et al.* Volcanism and the little ice age. *PAGES News* **16**, DOI: 10.22498/pages.16.2.22 (2008).
23. Toohey, M. & Sigl, M. Volcanic stratospheric sulfur injections and aerosol optical depth from 500 BCE to 1900 CE. *Earth Syst. Sci. Data* **9**, 809–831, DOI: https://doi.org/10.5194/essd-9-809-2017 (2017).
24. Khodri, M. *et al.* Tropical explosive volcanic eruptions can trigger El Niño by cooling tropical Africa. *Nat. Commun.* **8**, 1–13, DOI: 10.1038/s41467-017-00755-6 (2017).

25. Robock, A. Comment on No consistent ENSO response to volcanic forcing over the last millennium. *Science* **369**, DOI: 10.1126/science.abc0502 (2020).

26. PAGES2k Consortium. A global multiproxy database for temperature reconstructions of the Common Era. *Sci. Data* **4**, 170088, DOI: 10.1038/sdata.2017.88 (2017).

27. Diaz, H. F., Hoerling, M. P. & Eischeid, J. K. ENSO variability, teleconnections and climate change. *Int. J. Climatol.* **21**, 1845–1862, DOI: 10.1002/joc.631 (2001).

28. Predybaylo, E., Stenchikov, G., Wittenberg, A. T. & Osipov, S. El Niño/Southern Oscillation response to low-latitude volcanic eruptions depends on ocean pre-conditions and eruption timing. *Commun. Earth & Environ.* **1**, 1–13, DOI: 10.1038/s43247-020-0013-y (2020).

29. Vaccaro, A. *et al.* Climate Field Completion via Markov Random Fields: Application to the HadCRUT4.6 Temperature Dataset. *J. Clim.* **34**, 4169–4188, DOI: 10.1175/JCLI-D-19-0814.1 (2021).

30. Guillot, D., Rajaratnam, B. & Emile-Geay, J. Statistical paleoclimate reconstructions via Markov random fields. *The Annals Appl. Stat.* **9**, 324–352, DOI: 10.1214/14-AOAS794 (2015).

31. Hansen, J., Ruedy, R., Sato, M. & Lo, K. Global Surface Temperature Change. *Rev. Geophys.* **48**, DOI: 10.1029/2010RG000345 (2010).

32. Stevenson, S. *et al.* Volcanic Eruption Signatures in the Isotope-Enabled Last Millennium Ensemble. *Paleoceanogr. Paleoclimatology* **0**, DOI: 10.1029/2019PA003625 (2019).

33. Brady, E. *et al.* The Connected Isotopic Water Cycle in the Community Earth System Model Version 1. *J. Adv. Model. Earth Syst.* **11**, 2547–2566, DOI: 10.1029/2019MS001663 (2019).

34. Comboul, M. *et al.* A probabilistic model of chronological errors in layer-counted climate proxies: applications to annually banded coral archives. *Clim. Past* **10**, 825–841, DOI: https://doi.org/10.5194/cp-10-825-2014 (2014).

35. McGregor, S. *et al. The Effect of Strong Volcanic Eruptions on ENSO*, chap. 12, 267–287 (American Geophysical Union (AGU), 2020). DOI: 10.1002/9781119548164.ch12.

36. Emile-Geay, J., Cobb, K. M., Mann, M. E. & Wittenberg, A. T. Estimating Central Equatorial Pacific SST Variability over the Past Millennium. Part II: Reconstructions and Implications. *J. Clim.* **26**, 2329–2352, DOI: 10.1175/JCLI-D-11-00511.1 (2013).

37. Wilson, R. *et al.* Reconstructing ENSO: the influence of method, proxy data, climate forcing and teleconnections. *J. Quat. Sci.* **25**, 62–78, DOI: 10.1002/jqs.1297 (2010).

38. Toohey, M. *et al.* Disproportionately strong climate forcing from extratropical explosive volcanic eruptions. *Nat. Geosci.* **12**, 100–107, DOI: 10.1038/s41561-018-0286-2 (2019).
